# Extracellular heme recycling and sharing across species by novel mycomembrane vesicles of a Gram-positive bacterium

**DOI:** 10.1038/s41396-020-00800-1

**Published:** 2020-10-09

**Authors:** Meng Wang, Yong Nie, Xiao-Lei Wu

**Affiliations:** 1 College of Engineering, Peking University, 100871 Beijing, China; 2 Institute of Ocean Research, Peking University, 100871 Beijing, China; 3 Institute of Ecology, Peking University, 100871 Beijing, China

**Keywords:** Bacteria, Microbial ecology

## Abstract

Microbes spontaneously release membrane vesicles (MVs), which play roles in nutrient acquisition and microbial interactions. Iron is indispensable for microbes, but is a difficult nutrient to acquire. However, whether MVs are also responsible for efficient iron uptake and therefore involved in microbial interaction remains to be elucidated. Here, we used a Gram-positive strain, *Dietzia* sp. DQ12-45-1b, to analyze the function of its MVs in heme-iron recycling and sharing between species. We determined the structure and constituent of MVs and showed that DQ12-45-1b releases MVs originating from the mycomembrane. When comparing proteomes of MVs between iron-limiting and iron-rich conditions, we found that under iron-limiting conditions, heme-binding proteins are enriched. Next, we proved that MVs participate in extracellular heme capture and transport, especially in heme recycling from environmental hemoproteins. Finally, we found that the heme carried in MVs is utilized by multiple species, and we further verified that membrane fusion efficiency and species evolutionary distance determine heme delivery. Together, our findings strongly suggest that MVs act as a newly identified pathway for heme recycling, and represent a public good shared between phylogenetically closely related species.

## Introduction

Microorganisms naturally live in large communities, and are constantly challenged by lack of resources or environmental stimuli. To overcome this predicament, microbes form complex webs of ecological interactions with their neighbors, primarily via metabolic secretion and exchange [1, 2]. For instance, proteases, hydrolases, and quorum-sensing signal molecules are secreted through various secretion systems, thus assisting microbes to acquire nutrient and regulate community structures [3, 4]. Membrane vesicles (MVs) have recently been identified as type zero secretion systems [5], which are nano-sized spherical buds regularly released from microbial cellular membranes. Therefore, MVs are suitable for packing multi-molecule complexes and for delivering across long distances, and thus play critical roles in stress response, nutrition acquisition, host survival, and microbial interactions [6]. The cargoes packed in MVs are determined by their origination [7]. MVs are released from the outer membrane of bacterial cells, such as outer membrane vesicles (OMVs) from Gram-negative bacteria, and from the cytoplasmic membrane of cells, such as MVs from Gram-positive bacteria. Typically, OMVs are dominated by outer membrane proteins, while the cytoplasmic membrane proteins and cytoplasmic proteins are the primary contents of MVs.

The cargo of MVs is reflective of the composition of the producing cell, and changes in the cargo can alter the function of the MVs [8]. For example, MVs from *E. coli* under envelope stress contain multiple misfolded proteins, whose release enhances bacterial survival [9]. *Fibrobacter succinogenes* releases MVs packed with carbohydrate-active enzymes to primarily digest insoluble cellulose [10], while *Moraxella catarrhalis* secrets MVs loaded with UspA1/A2 virulence factors, which participate in host immune suppression [11]. Furthermore, MVs are used for intercellular communication, which is achieved through fusion to target cells. For example, DNA-carrying MVs are responsible for horizontal gene transfer [12]. In addition, due to their lipidic nature, MVs are highly suitable for delivery of hydrophobic signal molecule between cells, such as the quorum-sensing compound homoserine lactone [13, 14].

However, the aforementioned function studies mainly focused on Gram-negative bacteria OMVs, while the MVs functions from Gram-positive bacteria are less well studied [7, 15], being restricted to research on pathogens and mycobacteria [16–18]. Recently, some specific Gram-positive bacteria, including *Mycobacterium tuberculosis* and *Corynebacterium glutamicum*, were found to possess a novel cell wall structure consisting of an arabinogalactan layer covalently linked to the peptidoglycan layer, and a special outer membrane consisting of mycolic acids (mycomembrane) [19]. Whether these mycomembrane containing Gram-positive bacteria release MVs with diverse functions from this unique outer membrane remains unknown.

Iron is an obligate element for life because of its involvement in redox reactions and electron transfer [20]. Iron is almost insoluble at physiological pH (*K*_*sp*_ = 10^-58^), as a result of which it is extremely difficult for bacteria to sequester sufficient amounts of molecular iron [21]. To acquire iron ions under iron-limiting environmental conditions, bacteria secrete low-molecular-weight iron chelators called siderophores [22]. In addition, an important iron source for both pathogenic and environmental microbes is heme. The main heme sources for pathogenic microbes are hemoglobin and albumin in host [20]. In natural environments, hemoproteins are widely distributed and utilized by microbes. For instance, heme is the key prosthetic group of oxidoreductases involved in respiration, photosynthesis, ammonia assimilation, and dinitrogen fixation [23]. Furthermore, hemoproteins are widely found in natural microbial biofilms [24, 25]. Another study reported that nanomolar equivalents of iron (III) protophorphyrin IX-like compounds were found in natural estuarine waters and rivers [26].

To assimilate heme, bacteria exploit cell-surface heme-binding proteins, as well as a small number of hemophores, which bind heme extracellularly [27]. Gram-negative bacteria such as *Pseudomonas aeruginosa* and *Haemophilus influenzae* possess a conserved heme acquisition system that consists of several outer membrane receptors, the periplasmic TonB complex, and ABC transporter in the cytomembrane [22, 28]. Heme recognition and binding are conducted by sortase-linked proteins on the cell surface of Gram-positive bacteria, such as IsdH from *Staphylococcus aureus* and IsdX1/2 from *Bacillus anthracis* [29, 30]. These proteins contain NEAT (near transporter) domains, which are responsible for heme binding [31]. *Corynebacterium diphtheriae* utilizes a unique system for heme transport, which consists of HtaAB and HmuTUV. The cell-surface exposed heme-binding proteins HtaA and HtaB are lipoproteins that anchor to the cytomembrane. Their heme-binding mechanism is independent of the NEAT-binding domain [32, 33]. HtaA captures both free heme molecule and heme from hemoglobin. After binding, heme is subsequently transferred from HtaA to HtaB, and is further shuttled to the ABC transporter HmuTUV before degraded by heme oxygenase in cytoplasm [31].

Although the pathways responsible for heme assimilation have been identified, they are mostly heme-cell or hemoprotein-cell contact-dependent. Given that the concentration of heme or hemoprotein is very low in natural environments, direct heme-cell contact is presumably of low probability. Also, limited species secrete hemophores to acquire heme, which is therefore not a general pathway [22]. Whether bacteria universally acquire heme in a wide range remains unknown.

*Dietzia* sp. are high G + C content Gram-positive bacteria belonging to the order *Corynebacteriales*. One of their characteristics is the presence of mycomembrane containing mycolic acids. *Dietzia* sp. have been isolated from various extreme environments, such as Korean salted food, East African soda lake, Egypt desert, and plateau permafrost regions [34–37]. *Dietzia* sp. DQ12-45-1b was originally isolated from oil well produced liquid, where iron sources are very limited [38]. Here, we set out to investigate the structure of DQ12-45-1b MVs, and their roles in extracellular heme recycling and sharing. We showed that DQ12-45-1b releases MVs that originate from the mycomembrane. Furthermore, we showed that MVs capture and deliver heme in a *Dt*HtaA-dependent manner. Our results clearly demonstrate that MVs recycle extracellular heme, and suggest that MVs play critical roles in heme sharing within diverse microbial communities.

## Materials and methods

### Strains and culture conditions

*Dietzia* sp. DQ12-45-1b was cultured in GPY medium containing glucose 10 g/L, yeast extract 5 g/L, and tryptone 10 g/L at 30 °C. The defined minimal medium supplemented with 0.5% sodium acetate was used for iron-controlled culture (Na_2_HPO_4_·12H_2_O 17.9 g/L, NaH_2_PO_4_·2H_2_O 7.8 g/L, (NH_4_)_2_SO_4_ 5 g/L, KCl 5 g/L, MgSO_4_·7H_2_O 0.2 g/L, CaCl_2_ 0.05 g/L, ZnSO_4_·7H_2_O 0.1 mg/L, MnCl_2_·4H_2_O 0.03 mg/L, H_3_BO_3_ 0.3 mg/L, CoCl_2_·6H_2_O 0.2 mg/L, CuCl_2_·2H_2_O 0.01 mg/L, NiCl_2_·6H_2_O 0.02 mg/L, Na_2_MoO_4_·2H_2_O 0.03 mg/L, pH 8.0). To achieve iron-limiting and iron-rich culture conditions, the defined minimal medium supplemented with either 8 μM or 40 μM FeCl_3_ were used, respectively. For heme cultures, DQ12-45-1b was inoculated in defined minimal medium supplemented with 0.5% sodium acetate and 2 μM hemin (for clarity, hemin and heme were unified as heme herein). For genetic manipulation, *Escherichia coli* DH5α (Takara, Tokyo, Japan) was grown in Luria-Bertani (LB) medium at 37 °C. All strains containing a plasmid were grown in the appropriate antibiotic (kanamycin, 50 μg/mL; ampicillin, 100 μg/mL; streptomycin, 30 μg/mL).

For interspecific heme delivery experiments, multiple strains were cultured in LB medium to mid-exponential phase, then washed three times with PBS solution (pH 7.4; 8000 rpm, 5 min). These cell suspensions were then inoculated into minimal medium for culture (OD_600_ = 0.1). *Dietzia* genus (*D. natronolimnaea* HS-1, *D. maris* IMV-195, *D. cinnamea* P4, and *D. psychralcaliphila* ILA-1) were inoculated in defined minimal medium supplemented with 0.5% sodium acetate. The defined minimal medium supplemented with 0.5% glycerol and 0.2% Tween-80 was used for culture of *Corynebacterium glutamicum* ATCC13032, *Rhodococcus erythropolis* ATCC19369, and *Mycobacterium smegmatis* mc^2^155. *Pseudomonas fluorescens* CHA0, *Escherichia coli* DH5α, *Bacillus subtilis* ATCC 6051, *Bacillus cereus* ATCC 14579, and *Acinetobacter baumannii* ATCC19606 were grown in M9 minimal medium [39] without FeCl_3_ addition. *M. smegmatis*, *B. subtilis*, *B. cereus*, and *E*. *coli* were cultured at 37 °C, while all other species were grown at 30 °C.

### Gene knockout and complement

To generate Δ*DthtaA* and Δ*DthmuUV* mutant strains, a double homologous recombination method was used [40, 41]. Briefly, adjacent homologous fragments of *DthtaA* and *DthmuUV* (~600 bp) were PCR amplified. Next, the homologous fragments were fused to a selective streptomycin cassette by PCR. These fragments were then introduced into DQ12-45-1b::pJV-53 according to a method described previously [42]. After homologous recombination, target genes were replaced by the streptomycin cassette. The resulting DQ12-45-1b streptomycin-resistant strain was cultured in GPY-containing streptomycin. Here we designated the mutant strains as DQ12-45-1b Δ*DthtaA* and DQ12-45-1b Δ*DthmuUV*.

To generate mutant complements, the plasmid pNV-18-dsRed framework was used [43]. The plasmid was PCR-cloned without the dsRed region. Next, a DNA fragment containing either *DthtaA* or *DthmuUV* was PCR amplified starting with the DQ12-45-1b genome. pNV-18-*DthtaA* and pNV-18-*DthmuUV* were constructed using Hieff Clone^®^ Plus One Step Cloning Kit (Yeasen, Nanjing, China) according to the manufacturer’s instructions. The resulting recombinant plasmids were then transformed into *E. coli* DH5α. After extraction and desalting, the plasmids were electro-transformed into DQ12-45-1b Δ*DthtaA* and Δ*DthmuUV*, resulting in the complement strains DQ12-45-1b Δ*DthtaA*::*DthtaA* and DQ12-45-1b Δ*DthmuUV*::*DthmuUV*, respectively. Plasmid and genomic DNA extraction, PCR, and transformation of *E. coli* were performed using the standard methods described elsewhere [44]. Primers used are listed in Table S1.

### MVs isolation and purification

MVs were isolated from the DQ12-45-1b medium during the late-exponential phase. One-liter of culture was centrifuged to remove the majority of cells (8000 rpm, 15 min), before the supernatant was filtered through a 0.22 μm polyethersulfone membrane (Millipore, MA, USA) to exclude any residual cells. The filtrate was concentrated 20-fold using 100 kD Cross Flow UltraFiltration System (Sartorius, Goettingen, Germany). The concentrate was then centrifuged at 4 °C for 2 h at 200,000 × *g* (Beckman, CA, USA). The pellets were resuspended in Optiprep solution (Alere Technologies, Oslo, Norway) to a final concentration of 45% (*w/v*). The crude samples were then overlaid with a series of Optiprep gradient layers, with concentrations ranging from 40–10%. Samples were centrifuged at 200,000 × *g* for 6 h using swing bucket rotor SW 40Ti (Beckman). The vesicle fractions were collected and pelleted at 4 °C 200,000 × *g* for 2 h, and then washed twice with PBS buffer. The lipid content of MVs was determined by FM4-64 (Thermo Fisher, Waltham, America) as previously described [45].

### Microscopy

Transmission electron microscope (TEM) was used to analyze the morphology of cells and MVs. Cells and purified MVs were fixed with 2.5% glutaraldehyde at room temperature for 1 h. Then the cells and MVs were adsorbed on copper EM grids for 1 min, followed by negative staining with 1% uranyl acetate for 2 min and rinsed three times with double distilled water. Samples were analyzed at 80 kV using Hitachi HT7700. To observe the membranous origination of MVs, thin-section TEM was performed as previously described [46].

### Lipid analysis by TLC

Glycolipids from DQ12-45-1b cells and MVs were extracted and analyzed as previously described [47]. Briefly, cells and MVs were resuspended in 10 mL CHCl_3_–CH_3_OH (1:2) overnight at room temperature. Next, 10 mL CHCl_3_–H_2_O (1:1) were added to form two phases. The organic phase was collected and dried under nitrogen, and lipids were resuspended in 500 μL CHCl_3_. Samples were then spotted onto a Merck TLC Silica gel 60 plate and developed using CHCl_3_–CH_3_OH–H_2_O (65:25:4). The glycolipids were visualized by 0.2% anthranone. To extract total mycolic acids, MVs and cells were freeze-dried and then incubated with 2.8 mL ethylene glycol monomethyl ether and 0.4 mL 40% KOH per 10 mg biomass at 100 °C for 1 h. After cooling the samples to room temperature, 1 mL 20% H_2_SO_4_ was added, followed by three rounds of diethyl ether extraction. The resulting extraction liquor was washed three times with double distilled water, and then evaporated until dry. The resulting pellets were dissolved in 200 μL diethyl ether [48]. Samples were then developed in *n*-hexane-diethyl ether (4:1), and spots were visualized using 5% phosphomolybdic acid.

### Protein identification by LC−MS/MS

To identify the protein component of MVs, proteome detection and analysis was conducted as previously described [49]. Briefly, purified MVs from iron-limiting and iron-rich medium were collected. Protein samples were then separated and purified by SDS-PAGE. The samples were digested by trypsin (Promega, Madison, USA) and the resulting peptide fragments were loaded into LC-LTQ/Orbitrap-MS (Thermo Fisher Scientific). Protein annotation was generated against the DQ12-45-1b genome database (Genbank accession: GCA_009740915.1). Proteomes were quantified by comparing the spectrometric spectral peptides counts [50]. Proteins with more than three peptides counts were recorded.

### Heme binding and delivery measurement

To test whether *Dt*HtaA binds heme, we cloned *DthtaA* into pET-28a in *E. coli* BL21 for overexpression [44]. After 4 h expression by IPTG, cells were lysed by sonication and then centrifuged (6000 × *g*, 20 min, 4 °C). Next, *Dt*HtaA containing a His-tag in the supernatant was purified using Ni-NTA agarose (Qiagen, Dusseldorf, Germany) according to the manufacturer’s instructions. The *Dt*HtaA was incubated with 10 μM heme at 30 °C for 30 min and then purified by washing [44]. Simultaneously, a TetR family repressor AlkX from DQ12-45-1b, which is involved in the expression regulation of alkane hydroxylase [51], was purified and incubated with heme as negative control. The purified protein solutions were scanned from 350 to 500 nm using a microplate reader (Molecular Devices, Sunnyvale, USA). Similarly, to test whether *Dt*HtaA binds the heme of hemoproteins, 5 μM *Dt*HtaA was incubated with 50 μM cytochrome P450, lignin peroxidase, cytochrome *c*, and catalase (Sigma-Aldrich), respectively, at 30 °C for 2 h. Lastly, the *Dt*HtaA was purified as before and the OD values were recorded. Unincubated *Dt*HtaA was used as control.

To assess the capacity of MV mediated heme delivery, MVs from iron-limiting cultures were isolated and purified as before, followed by incubation with excess heme (50 μM) for 2 h at 30 °C. The resulting heme-incubated MVs were washed with PBS buffer 3 times at 200,000 × *g* for 1 h. Then, 100 μg MVs were filter sterilized and added to DQ12-45-1b as the sole iron source, and cultured at 30 °C for 72 h. The growth curve of DQ12-45-1b was recorded to test whether MV mediated heme delivery occurred. Simultaneously, DQ12-45-1b cultured without iron source and with 100 μg unincubated MVs were used as negative controls. DQ12-45-1b cultured with 2 μM heme as sole iron source was used as positive control and the maximal OD_600_ was calibrated as 100%. To check whether free heme is released from heme-incubated MVs and absorbed by DQ12-45-1b, we incubated the heme-incubated MVs solution at 30 °C for 4 days. Lastly, MVs were excluded by ultrafiltration (1000 kD), and the filtrate was incubated with DQ12-45-1b as sole iron source at 30 °C for 72 h, while monitored for growth.

To test whether MVs capture heme from hemoproteins, MVs from iron-limiting cultures were incubated with 50 μM cytochrome P450, lignin peroxidase, cytochrome *c*, and catalase at 30 °C for 2 h, followed by three ultracentrifugation washings (200,000 × *g*, 1 h). Next, 100 μg of the resulting MVs were incubated with DQ12-45-1b in defined minimal medium, and the OD_600_ was recorded. DQ12-45-1b cultured with 100 μg unincubated MVs and 2 μM heme were used as negative and positive controls, respectively. Simultaneously, hemoprotein as sole iron source was used to culture DQ12-45-1b as positive control (cytochrome P450, 2 μM; lignin peroxidase, 2 μM; cytochrome *c*, 2 μM; and catalase, 0.5 μM). For interspecific heme delivery, 100 μg heme-incubated MVs or unincubated MVs were used as the sole iron source to culture *Dietzia* species, *C. glutamicum*, *R. erythropolis*, *M. smegmatis*, *P. fluorescens*, *E*. *coli*, *B. subtilis*, *B. cereus*, and *A. baumannii*. The growth curves were measured and the maximal OD_600_ readings were recorded. To obtain positive controls, each strain was cultured in their corresponding minimal medium amended with 2 μM heme and their growth status was recorded as 100%.

### Fusion efficiency measurement

To study whether MVs fused to target cells, a previously published fluorescence labeling method was modified [14, 52]. The heme-incubated MVs were harvested and incubated with FM4-64 (3.3 μg/mL in PBS buffer at 37 °C for 10 min). The labeled MVs were then washed by ultracentrifugation (200,000 × *g*, 1 hour, twice) and incubated with 1.0 OD_600_ of different cells in PBS buffer for 1 h. The incubated cells were then washed three times (8000 rpm, 2 min) with PBS buffer to remove the free MVs. The fluorescence intensity of cells was measured by a microplate reader with excitation at 509 nm and emission at 750 nm and analyzed by fluorescence microscope (Leica DM6000). The fluorescence intensity of DQ12-45-1b cells after incubation with labeled MVs was calibrated as 100%.

### Computer analysis

Protein domains were analyzed using the NCBI conserved domain database [53]. Sequences of the aforementioned species 16S rDNA were downloaded from NCBI. Sequence alignment and phylogenetic analysis were performed using MEGA6 [54]. Protein signal peptides were predicted using PSORTb [55]. To predict mycomembrane proteins in DQ12-45-1b, the β-strands strategy for *M. tuberculosis* mycomembrane protein calculation was applied [56]. The Genbank accession numbers of *Dt*HtaA, *Dt*HtaB, *Dt*HmuT, *Dt*HmuU, and *Dt*HmuV are QGW23845.1, QGW23844.1, QGW23840.1, QGW23841.1, and QGW23842.1, respectively. The protein dataset of MVs were deposited at figshare (10.6084/m9.figshare.12465311).

## Results

### The Gram-positive bacterium DQ12-45-1b releases mycomembrane vesicles under iron-limiting conditions

**Fig. 1:**
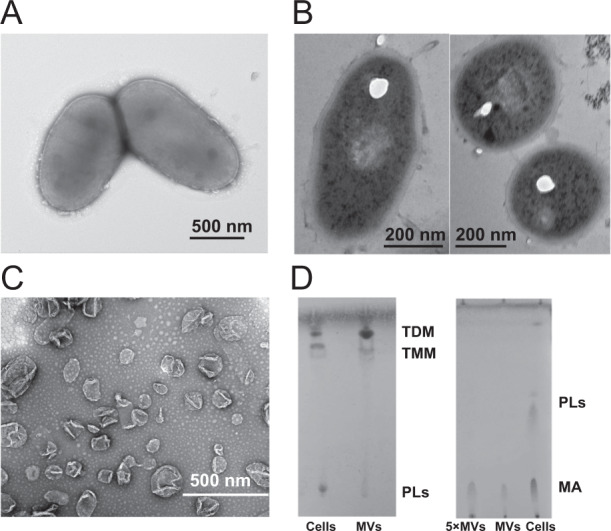
Spontaneously secreted MVs of *Dietzia* sp. DQ12-45-1b. A. Negative-staining micrograph of DQ12-45-1b cells by TEM. B. Ultrathin section observation of DQ12-45-1b cells by TEM. Sections with thickness of 50–70 nm were achieved. C. TEM micrograph of purified MVs from density gradient centrifugation. D. Glycolipids and mycolic acids identified in cells and MVs. Glycolipids were visualized using anthrone, and mycolic acids were visualized using phosphomolybdic acid. MA mycolic acids, PLs phospholipids, TDM trehalose dimycolate, TMM trehalose monomycolate.

In order to evaluate whether DQ12-45-1b generates MVs, we analyzed the morphology of DQ12-45-1b cells from late-exponential phase in iron-limiting medium using TEM. As shown in Fig. 1a, b, we observed surface protrusions on the cells of DQ12-45-1b, with the thin-section TEM of cells showing that the continuous membrane protrudes from the mycomembrane, with a clear boundary at the relevant inner membrane location. To investigate whether these protrusions are released from cells and form MVs, we collected DQ12-45-1b cultures grown under iron-limiting conditions during late-exponential phase. After ultracentrifugation, reddish pellets formed at the bottom of the tubes. The reddish pellets showed vesicular structures, including both tube-like and spherical structures with measurements like the cell-surface protrusions observed under TEM (Fig. 1c). Moreover, we failed to recover these vesicular structures from dead cells, suggesting that these structures are actively secreted by live cells rather than being formed by cell debris (Fig. S1). Therefore, we considered these structures to be MVs that are actively released by DQ12-45-1b.

To confirm whether these MVs are released from the mycomembrane, we characterized their lipid contents using TLC (thin layer chromatography). The cell wall of *Dietzia* and related species such as *Mycobacterium* have a special mycomembrane consisting of mycolic acids [57]. We found trehalose dimycolate (TDM), trehalose monomycolate (TMM), and mycolic acids, which are the main components of the mycomembrane, in both MVs and the intact cells. However, phospholipids, which are the main component of the inner membrane, were only identified in intact cells (Fig. 1d). Therefore, these findings suggest that the MVs of DQ12-45-1b represent mycomembrane vesicles (mMVs).

**Fig. 2:**
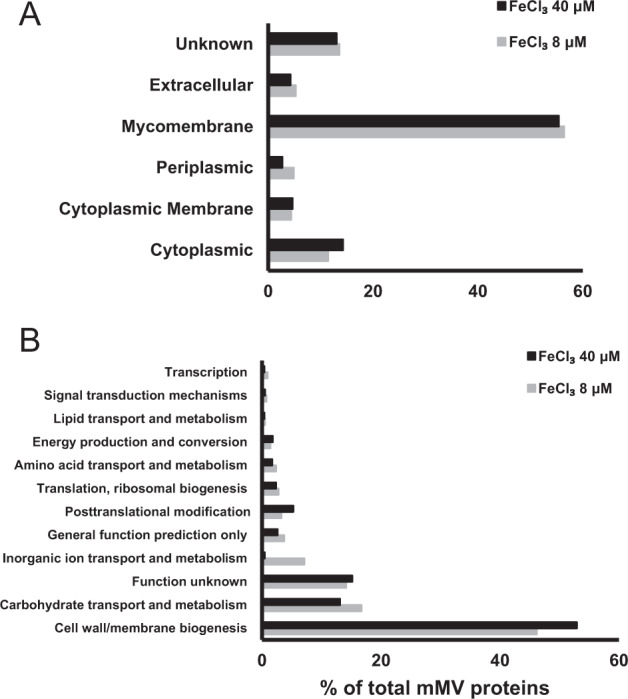
Comparative proteomic analysis of DQ12-45-1b mMVs. Subcellular location of mMVs proteins were predicted and analyzed as described in the “Materials and methods”. A. The abundance ratio of proteins with different subcellular locations. B. Function annotation of mMV proteins from iron-limiting (8 μM) and iron-rich conditions (40 μM). Function annotation was conducted against the COG database. Protein abundance was obtained by proteomic analysis.

We identified and analyzed the proteomes of mMVs from both iron-limiting and iron-rich cultures using LC–MS/MS (Table S2). Both mMV proteomes are dominated by mycomembrane proteins, which accounts for more than 50% of the total peptide counts (Fig. 2a). This result was in accordance with the protein features from Gram-negative bacteria OMVs [58], further supporting the idea that MVs of DQ12-45-1b constitute mycomembrane. In addition, to investigate the production of mMVs from the same volume of iron-limiting and iron-rich cultures, we collected mMVs and incubated them with FM4-64 at 37 °C for 10 min [52]. By detection fluorescence signal of mMVs with excitation at 509 nm and emission at 750 nm, we found that fluorescence intensity of mMVs from iron-limiting culture is threefold of that from iron-rich culture (Fig. S2), suggesting that mMVs represent a strategy of DQ12-45-1b to adapt to limited supply of iron in its environment.

### Novel heme-binding protein *Dt*HtaA: annotation and functions

In order to investigate the potential iron-regulated roles of mMVs, we compared the mMV protein profiles from iron-limiting and iron-rich cultures. As shown in Fig. 2b, our analysis resulted in classification of ~97% mMV proteins into 12 categories based on COG annotation. The most abundant orthologous groups encode for cell wall/membrane biogenesis, followed by carbohydrate transport and metabolism, and “function unknown”, together accounting for more than 76% of the total proteins. We further compared the expression levels of proteins in mMVs from iron-limiting and iron-rich cultures (Table 1, Fig. S3). Remarkably, proteins involved in iron assimilation are upregulated in mMVs from iron-limiting cultures, such as siderophore binding protein ORF2149, as well as two heme transport-associated domain containing proteins, ORF1247 and ORF1248. Under iron-limiting conditions, expression of ORF1248 and ORF1247 are upregulated by over 50-fold and 20-fold, accounting for 4.2 and 0.9% of the total mMV proteins, respectively. This result was consistent with greater mMV production observed under iron-limiting conditions, which suggested that mMVs from iron-limiting conditions is involved in iron transfer.

**Table 1:** Significantly changed proteins between mMVs from iron-limiting and iron-rich conditions.

			Abundance (%)			
	log_2_FC	*p*	mMVs iron rich	mMVs iron limiting	Gene product	Annotation
ORF2312	−3.10	0.019	0.03	0.01	InfC	Translation initiation factor IF-3
ORF2977	−3.72	0.025	0.04	0.01	LeuD	3-Isopropylmalate dehydratase
ORF1247	4.38	0.005	0.05	0.95	Hypothetical protein	HtaA domain detected
ORF1248	5.71	0.042	0.08	4.21	Hypothetical protein	HtaA domain detected
ORF1212	1.89	0.031	0.12	0.43	PstS	ABC-type phosphate transport system
ORF2149	2.32	0.040	0.20	0.99	FhuD	Iron-siderophore ABC transporter
ORF4534	1.26	0.005	0.43	1.04	PhoD	Alkaline phosphatase D
ORF5111	0.79	0.015	1.58	2.74	Lipase	Secretory lipase

Proteins with fold change > 1.5 or < 0.75 and *p* < 0.05 were analyzed.

FC fold change.

In order to predict the specific function of mMVs under iron-limiting conditions, we performed bioinformatic analysis against NCBI non-redundant protein database. As shown in Fig. S4, the two ORFs ORF1247 and ORF1248 locate adjacent to three heme ABC transporter proteins (ORF1240/1241/1242). The gene arrangement is similar to that found for the HtaAB-HmuTUV gene cluster of *Corynebacterium diphtheriae* [32], whose functions were previously identified as heme binding and transport. However, the ORF1248 found in DQ12-45-1b shares only 22.9% identity and 39% query coverage when compared to *C. diphtheriae* HtaA. Similarly, ORF1247 shares only 29.2% identity and 21% query coverage compared to *C. diphtheriae* HtaB. Moreover, ORF1248 contains a single HtaA conserved domain, while the *C. diphtheria* version possesses two HtaA conserved domains (Fig. S4). Because of the low identities to the known HtaA and HtaB associated proteins, ORF1248 and ORF1247 represent novel HtaA- and HtaB-like proteins, which we therefore named *Dt*HtaA and *Dt*HtaB, respectively.

**Fig. 3:**
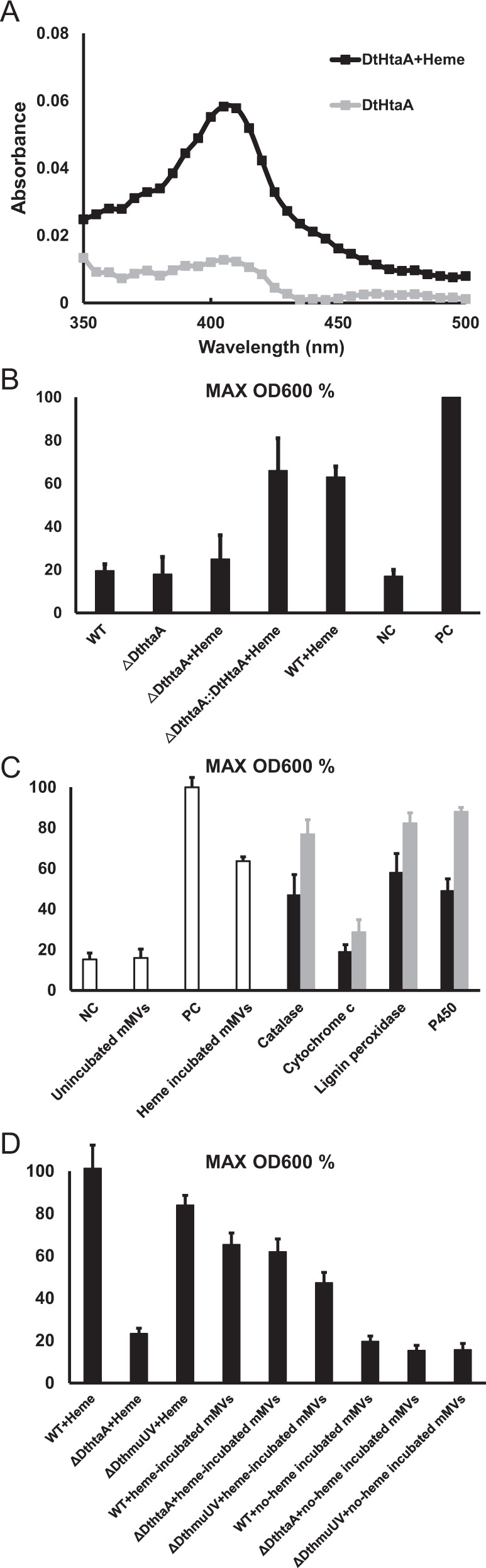
*Dt*HtaA in mMVs participates in heme binding and delivery. A. Purified *Dt*HtaA bound heme in vitro. *Dt*HtaA was incubated in 10 μM heme solution for 30 min. Excess heme was washed out and the protein solution was scanned from 350-500 nm. *Dt*HtaA without heme addition was used for comparison. One peak characteristic of heme was detected at 410 nm. B. *Dt*HtaA residing inside mMVs is responsible for heme binding and delivery. One-hundred micrograms of heme-incubated mMVs from wild type (WT), Δ*DthtaA*, and Δ*DthtaA*::*DthtaA* were added to DQ12-45-1b culture, and the maximum OD_600_ was recorded. PC positive control, DQ12-45-1b cultured with 2 μM heme as sole iron source (calibrated as 100%). NC negative control, DQ12-45-1b cultured without iron. C. mMVs capture and deliver heme from environmental hemoproteins. Hemoproteins were incubated with 100 μg mMVs at 30 °C for 2 h. mMVs were then washed and added to DQ12-45-1b culture as sole iron source, and the maximal OD_600_ was recorded (black). The growth of DQ12-45-1b using hemoprotein as sole iron source was recorded (gray). PC positive control, DQ12-45-1b cultured with 2 μM heme as sole iron source (calibrated as 100%). NC negative control, DQ12-45-1b cultured without iron. D. Involvement of *Dt*HtaA and *Dt*HmuUV in free heme and heme-incubated mMVs related heme utilization. The strain DQ12-45-1b, DQ12-45-1b Δ*DthtaA,* and DQ12-45-1b Δ*DthmuUV* were cultured in defined minimal medium amended with 2 μM heme, 100 μg no-heme-incubated mMVs and 100 μg heme-incubated mMVs, respectively. After cultured for 72 h at 30 °C, the strains’ maximal growth at OD_600_ were recorded. The growth of DQ12-45-1b in free heme was calibrated as 100%. All mMVs used in this experiment were isolated from DQ12-45-1b cultures at iron-limiting conditions. Error bar represents three independent replicates.

To determine whether *Dt*HtaA binds heme, we incubated 1 μM purified *Dt*HtaA with 10 μM heme at 30 °C for 30 min. After washing out excess heme, purified *Dt*HtaA was obtained using Ni-affinity chromatography. We found *Dt*HtaA exhibited maximum absorbance at 410 nm (Fig. 3a), which represents the characteristic peak of heme. Meanwhile, there was no peak detected in the case of repressor protein AlkX (Fig. S5). Therefore, this result indicated that *Dt*HtaA is able to bind heme. In addition, to test whether *Dt*HtaA captures heme from environmental hemoproteins, we incubated 5 μM purified *Dt*HtaA with 50 μM of four hemoproteins: cytochrome P450, lignin peroxidase, cytochrome *c*, and catalase, at 30 °C for 2 h, respectively, and purified *Dt*HtaA as described above. As shown in Fig. S6, we detected the characteristic heme absorbance peak around 410 nm of *Dt*HtaA that incubated with cytochrome P450, lignin peroxidase, and catalase. In addition, we failed to detect significant binding of heme from cytochrome *c*. We also investigated whether the heme was released by the hemoproteins themselves, however, no significantly heme release was detected (data not shown). Together, these results showed that *Dt*HtaA captures specific hemes available in the environment.

In order to identify the subcellular location of *Dt*HtaA, we performed western blotting and immuno-electron microscope of the membrane factions [59]. As shown in Fig. S7, *Dt*HtaA locates in the mycomembrane of DQ12-45-1b and the surface of mMVs with similar abundance. These results indicated that *Dt*HtaA in mMVs locates in the same membrane as its cellular homolog. This also suggests that *Dt*HtaA maintains its function once released with mMVs into the extracellular space.

### mMVs acts as vehicles of *Dt*HtaA to deliver and transport heme into DQ12-45-1b

To analyze whether mMVs mediate heme delivery, we harvested mMVs from iron-limiting cultures and incubated them with 50 μM heme for 2 h at 30 °C. Under iron-free conditions, we found that adding heme-incubated mMVs to the DQ12-45-1b, their growth rates significantly increased (Fig. 3b). In contrast, incubation of cells with heme-free mMVs failed to increase growth rates. Together, the results indicated that mMVs bind extracellular heme and transfer it to DQ12-45-1b cell as an iron source. To verify whether heme delivery occurs in a *Dt*HtaA-dependent manner, we harvested the mMVs from Δ*DthtaA* cells under iron-limiting conditions and incubated them with heme as described above. As shown in Fig. 3b, mMVs of Δ*DthtaA* cells failed to facilitate the transfer of mMV-associated heme into target cells. Next, we complemented Δ*DthtaA* cells and the released mMVs supported the growth of DQ12-45-1b by delivering extracellular heme to the cells. These results indicated that *Dt*HtaA is essential for extracellular heme delivery by mMVs.

In addition to our binding studies for free heme, we tested whether mMVs bind heme molecules from environmental hemoproteins and support the growth of DQ12-45-1b as before. To this end, we incubated mMVs from iron-limiting cultures with the above hemoproteins for 2 h at 30 °C and purified the mMVs again. As shown in Fig. 3c, we found that mMVs incubated with either cytochrome P450, catalase, or lignin peroxidase significantly supported cell growth, compared to cells in the negative control samples. mMVs were not able to extract heme from cytochrome *c*, while DQ12-45-1b cells showed slight growth with cytochrome *c* as sole iron source (*p* = 0.02). This result indicated that although *Dt*HtaA dominates heme delivery, there still are some unknown proteins involved in heme assimilation from hemoproteins (Fig. S8). Next, we analyzed the heme types from these extractable hemoproteins and found that mMVs primarily bind heme from heme *b* type hemoproteins. Together, these results suggested that mMVs act as vehicles for *Dt*HtaA, and bind specific heme to support bacterial growth. Therefore, we propose that this process constitutes as a hitherto unknown pathway for extracellular heme recycling.

Two mechanisms might explain how DQ12-45-1b captures heme via mMVs. One feasible option is that the heme-binding mMVs release heme into the extracellular surroundings, from where the heme molecules are then absorbed by the cells. Alternatively, the heme-binding mMVs specifically attach or adjoin to the cell surface and the heme molecules are subsequently transported or jump to cells. To test which of these two mechanisms is used, we incubated the heme-binding mMVs by themselves at 30 °C for 4 days. Next, we separated and excluded mMVs by ultrafiltration. The filtrate was incubated with DQ12-45-1b as the sole iron source, and no significant growth was observed. This result suggests that the iron source for growth of DQ12-45-1b is directly from mMVs rather than the free heme molecules that released from mMVs. Therefore, we hypothesized that mMVs bind heme in the extracellular surroundings before the heme iron is absorbed through an mMV-dependent mechanism.

To test whether heme delivery of mMVs occurs in an HtaAB-HmuTUV-dependent manner, we constructed an ORF1241 and ORF1242 mutant strain Δ*DthmuUV*. Next, we measured whether *Dt*HtaA and *Dt*HmuUV are involved in heme uptake from heme-incubated mMVs. As shown in Fig. 3d, when we used free heme molecules as sole iron source, the maximal growth of DQ12-45-1b Δ*DthtaA* and Δ*DthmuUV* decreased by 76.4% and 16%, respectively. This result indicated that HtaAB-HmuTUV is involved in utilizing extracellular free heme. In addition, when we used heme-incubated mMVs as sole iron source, only the Δ*DthmuUV* cells exhibited reduced growth compared to wild-type strain. In contrast, the mutant strain Δ*DthtaA* showed little reduction in its maximal growth (*p* = 0.52). Together, these results suggested that the *Dt*HmuUV pathway is involved in utilizing both extracellular free heme and heme sequestrated in mMVs. However, the cellular *Dt*HtaA only participates in utilizing extracellular free heme. Moreover, when the heme-incubated mMVs were used as sole iron source, Δ*DthmuUV* still showed significant growth compared with negative control. On the basis of these results, we propose that the HtaAB-HmuTUV pathway is not the only pathway to absorb heme sequestrated in mMVs (Fig. S9).

### Interspecific mMV delivery

**Fig. 4:**
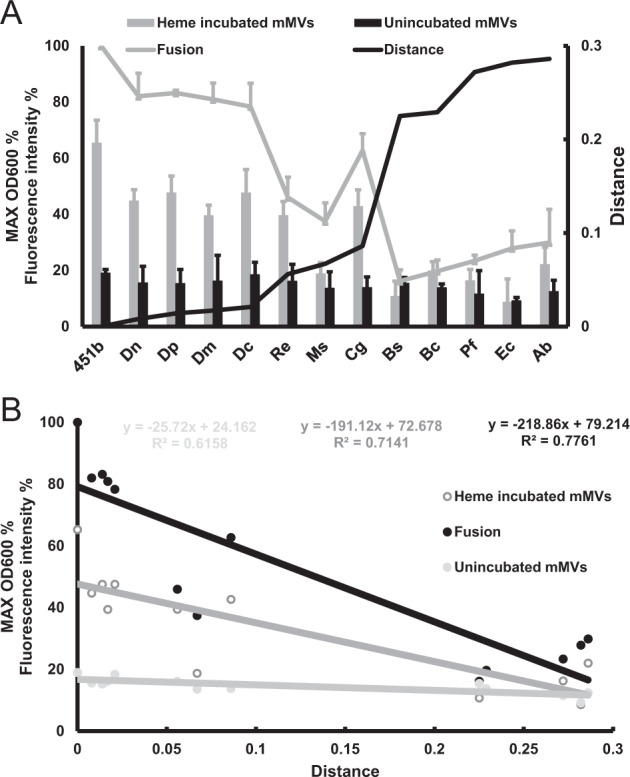
mMVs mediate interspecies heme delivery. A. mMVs mediated interspecific heme delivery is a function of fusion efficiency and homology distance. The heme-incubated mMVs and heme-free (unincubated) mMVs were added to multiple species as the sole iron source, and the maximal OD_600_ was recorded. The homology distance was calculated by MEGA6. The fusion efficiency was measured by FM4-64-based membrane labeling as described in the “Materials and methods”. The membrane fusion efficiency of heme-incubated mMVs to DQ12-45-1b was considered as 100%. To calibrate the growth of different strains, the maximal growth at the optimal conditions of each bacterium was used as 100%. 45-1b, *D*. sp. DQ12-45-1b, Dn *D. natronolimnaea*, Dp *D. psychralcaliphila*, Dm *D. maris,* Dc *D. cinnamea*, Re *R. erythropolis,* Ms *M. smegmatis,* Cg *C. glutamicum,* Bs *B. subtilis,* Bc *B. cereus,* Pf *P. fluorescens,* Ec *E. coli*, Ab *A. baumannii*. Error bar represents three independent replicates. B. Fitting analysis of homology distance, heme delivery, and fusion efficiency.

To investigate whether mMVs from DQ12-45-1b deliver heme to cells of a second species, we chose 12 strains belonging to the orders *Corynebacteriales*, *Pseudomonadales*, *Bacillales*, and *Enterobacterale* to assay the heme transport efficiency. First, we cultured these strains using heme-incubated mMVs as the iron source, with unincubated mMVs (heme-free) as negative controls. When using the heme-incubated mMVs as the iron source, we found that growth of *Dietzia* strains, *C. glutamicum*, and *R. erythropolis* was significantly greater (*p* < 0.05), when compared with the respective controls of heme-free mMVs (Fig. 4a). The strains from the genus *Dietzia* exhibited delivery efficiencies ranging from 39.4 to 47.6%, compared with the free heme as iron source, which were much higher than other strains. These results indicated that the mMV-dependent delivery of heme occurs across species. Importantly, the efficiency of heme delivery appears to depend on the phylogenetic relationship between donor and acceptor strains. Through calculation of the phylogenetic distances between the tested species and DQ12-45-1b, we found that evolutionary distance and efficiency of heme delivery are negatively correlated (Fig. 4b).

To identify whether the interspecific heme delivery depends on membrane fusion between mMVs and recipient cells, we determined the corresponding efficiency for membrane fusion using FM4-64 labeled heme-mMVs. As shown in Fig. 4, fusion efficiencies mimicked heme delivery efficiencies, with the strains from the genus *Dietzia* showing the highest fusion efficiencies, followed by *C. glutamicum* and *R. erythropolis*. The microscopic images of recipient cells further reflected that cells with higher fusion efficiencies exhibited more fluorescent cells and stronger intensities (Fig. S10). Moreover, the heme delivery efficiency was linearly correlated to the fusion efficiency of mMVs (*R*^2^ = 0.878, Fig. S11). Together, these results suggested that mMV fusion efficiency determines the interspecific utilization efficiency of heme sequestrated in mMVs. In addition, both heme delivery efficiency and mMV fusion efficiency were correlated to the phylogenetic distances (*R*^2^ > 0.7, Fig. 4b). From these results, we deduced that the species evolutionary distance influences the interspecific heme delivery efficiency. Despite of the random attachment exists, these findings demonstrated that mMVs are recruited by currently unknown mechanisms that slightly vary between strains.

## Discussion

We showed that mMVs of a Gram-positive bacterium deliver heme-iron directly into cells and acts as public goods under iron-depleted conditions. Our findings clearly demonstrate that MVs of *Dietzia* sp. DQ12-45-1b originate from the mycomembrane. DQ12-45-1b spontaneously produces mycomembrane vesicles, and these mMVs are characterized by lipids TDM, TMM, and mycolic acids, which are the major components of mycomembrane. In addition, we directly observed the derivation of mMVs by ultrathin section TEM micrographs. However, the mMVs of DQ12-45-1b appear to differ from those of other Gram-positive bacteria in various aspects. Previous studies reported that MVs are secreted from cytomembrane of Gram-positive bacteria, such as *Bacillus*, *Streptococcus*, and *Mycobacterium* [7, 17]. It is not unusual that the reported MVs of Gram-positive bacteria, which lack an outer membrane structure, originate from the cytomembrane. However, recent studies have reported that the cell wall of *Mycobacterium* and related species differ in composition from other Gram-positive bacteria [59, 60]. In the cell wall of *Mycobacterium*, an arabinogalactan layer and a special mycomembrane consisting of mycolic acids is present. Although these mycobacteria possess a mycomembrane structure, they release cytomembrane vesicles. The cell wall structure between mycobacteria and DQ12-45-1b is homologous, but the origination of MVs is different. The major difference of cell wall structure is that the aliphatic chain length of mycolic acids (ACLM) in DQ12-45-1b is much shorter than that in mycobacteria [61]. The ACLM in *Dietzia* sp. is 30–38 [61], while it is 70–90 in *Mycobacterium* sp [62]. Moreover, another previous study reported that TDM isolated from *Corynebacterium* sp. with ACLM ranging from 22 to 38 forms liposomes, while TDM from *Mycobacterium* sp. showed no such trait [63]. Therefore, we deduce that the vesicles originating from the mycomembrane may be formed by Gram-positive bacteria containing short chain mycolic acids. Nonetheless, how the mycomembrane detaches from arabinogalactan, or whether the peptidoglycan and arabinogalactan are also presented in mMVs remains unknown. Because of the special mycomembrane structure of the vesicles, the DQ12-45-1b mMVs contain high abundance of mycomembrane proteins, suggesting their novel functions.

We showed here that under iron-limiting conditions, *Dt*HtaA is one of the most abundant mycomembrane proteins in the mMVs of DQ12-45-1b. Previous reports have shown that the cell-surface exposed HtaA both binds free heme and captures heme from hemoglobin [33]. In DQ12-45-1b, *Dt*HtaA distributes both at the cellular mycomembrane and mMVs with similar abundance, which indicates that *Dt*HtaA is randomly packaged into mMVs. In addition, *Dt*HtaA that associated with mMVs distributes much broader than the cell-surface homolog with the free diffusion of mMVs [64], which assists cells for wide-range heme acquisition, especially when the extracellular heme is scarce and the motility of cells is limited. Similarly, siderophore-based iron ion binding by MVs has previously been reported for *Mycobacterium* [46]. Compared with soluble ferric ion that is bound by siderophores, extensive heme sources are available in natural environments, such as oxidoreductase released from cells, hemoglobin from animal blood and bodies, and photosynthesis-related proteins. Therefore, *Dt*HtaA-mediated delivery of heme from the environment by mMVs may represent a general phenomenon in nature. Here we concluded that *Dt*HtaA extracts the noncovalently bound heme *b*, as present in cytochrome P450, lignin peroxidase, and catalase, rather than the covalently attached heme *c* [65]. Therefore, the *Dt*HtaA captures heme, but with specificity to some environmental hemoproteins. However, the specific heme-binding capacity of *Dt*HtaA and its potential interaction with hemoproteins are currently unknown. Our study, for the first time, demonstrated that heme is a previously underestimated source of iron that can be recycled by mMVs.

We showed in this study that mMVs act as public goods shared both intraspecifically and interspecifically. Recent studies reported that extracellular siderophores are shared between phylogenetically closely related species, which typically express homologous siderophore system [66]. This is in agreement with our findings, except that the homologous heme transport system in recipient cells is not essential for heme delivery. At least two pathways are involved to recover extracellular heme carried by mMVs. First, heme is transferred from mMVs into cells through a *Dt*HmuUV pathway, independent of *Dt*HtaA in the recipient cells. Therefore, species with no *Dt*HtaA homologs may also benefit from the heme loaded mMVs. However, the Δ*DthmuUV* strains still exhibit significant heme uptake ability from mMVs (Fig. 3D), and there was no significant influence on fusion efficiency of mMVs to Δ*DthmuUV* cells (Fig. S9). In addition, comparative genomic analysis showed that species harboring no *Dt*HmuUV homologs still exhibit heme uptake ability through mMVs (unpublished data). We conclude from these results that *Dt*HmuUV is involved in heme delivery from mMVs, though it may not be essential. Second, mMVs-dependent heme delivery may occur after membrane fusion between mMVs and the phylogenetically closely related species, independent of *Dt*HmuUV pathway. The membrane compatibility of species may determine the fusion between mMVs and cells. The ACLM in *Dietzia*, *Corynebacterium*, and *Rhodococcus* are all among 28–46, which means their mycomembrane thickness is comparable (~5 nm) [67]. These homologous membrane constituents and thickness may result in high probability of membrane fusion. However, although *M. smegmatis* shares close homolog distance with DQ12-45-1b, the ACLM in *Mycobacterium* is 70–90, which leads to a much thicker mycomembrane (~8 nm) [68]. On the basis of these findings, we propose that the difference of membrane thickness is the major barrier for mMV fusion to *M. smegmatis* [52]. As for Gram-negative bacteria, whose outer membrane consists of lipopolysaccharides, the difference of membrane constituents may hinder mMV fusion. However, whether mMV fusion with the cell is essential for heme recruitment remains to be elucidated, and other processes such as endocytosis may also be involved.

**Fig. 5:**
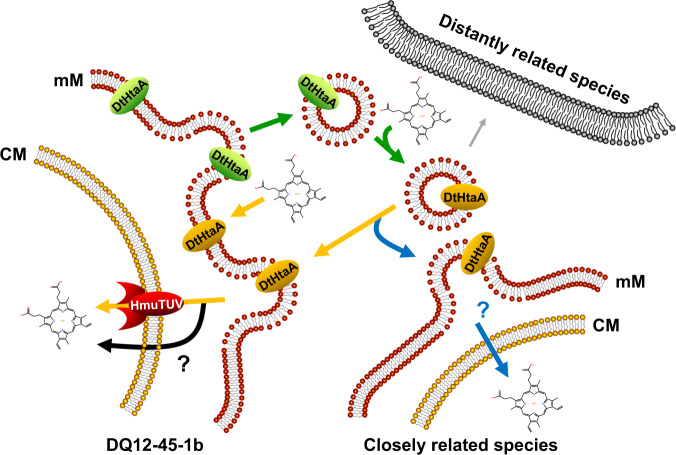
A model for *Dt*HtaA-mediated heme delivery by mMVs. DQ12-45-1b secreted mMVs that loaded with *Dt*HtaA under iron-limiting condition. After extracellular heme binding, the mMVs: (i) are recycled by DQ12-45-1b through membrane fusion, followed by transport via HmuTUV or other transporters located on the cytomembrane; (ii) fuse to phylogenetically closely related species with similar membrane thickness and constitutes, followed by heme release and transport into cytoplasm by unknown mechanisms; (iii) are less shared by other cells due to the related phylogenetically distantly homology or membrane difference. CM, cytomembrane. mM, mycomembrane, which was simplified as red bilayer here.

In natural ecological niches, microbes exist as aggregated communities, and public goods such as siderophores may benefit the entire community; alternatively, they might act as tools driving competitive exclusion [69]. It has been previously reported that MVs are shared by the community in diverse ecosystems. For instance, MVs from *Bacteroidales* in human gut microbial ecosystem contained diverse polysaccharides hydrolases. These MVs function cooperatively to extracellularly degrade polysaccharides to achieve syntrophic interactions in *Bacteroidales* [70]. In this work, we showed that mMV-dependent heme delivery may mainly benefit phylogenetically closely related species, which is a kin-selective benefit (Fig. 5). The mMV-dependent heme delivery might also improve the competitiveness of slow growing bacteria in resource limited environments. The released mMVs sequester the limited environmental iron resources and act as an iron repository for those slow growing bacteria, which inhibits depletion of public iron sources by fast growing bacteria, subsequently maintaining community stability.

In summary, we identified a novel type of mMVs released by a Gram-positive bacterium, and proved the underestimated roles of MVs in extracellular heme recycling and sharing. Therefore, the nutrient sequestered in MVs should be regarded as potential community regulators. Although further work is necessary to identify the mechanisms for mMV recruitment and fusion, our work provides novel insights into the understanding of iron utilization and strategies for maintaining the stability of a bacterial community surviving in an iron-limiting environment.

## Supplementary information

Supplementary material files

Figure S1

Figure S2

Figure S3

Figure S4

Figure S5

Figure S6

Figure S7

Figure S8

Figure S9

Figure S10

Figure S11

Table S1

Table S2 (pdf version)
