# Supplementary material for: Extracellular heme recycling and sharing across species by novel mycomembrane vesicles of a Gram-positive bacterium
Source: ISME J. 2020 Oct 9;15(2):605–17. doi: 10.1038/s41396-020-00800-1 (PMC8027190; doi:10.1038/s41396-020-00800-1)
Supplement: Supplementary file 1 — Supplementary material files [file 41396_2020_800_MOESM1_ESM.docx]

**Extracellular heme recycling and sharing across species by novel mycomembrane vesicles of a Gram-positive bacterium**

**Supplementary materials**

Meng Wang^1^, Yong Nie^1*^ and Xiao-Lei Wu^1,2,3*^

^1^ College of Engineering, Peking University, Beijing 100871, China

^2^ Institute of Ocean Research, Peking University, Beijing 100871, China

^3^ Institute of Ecology, Peking University, Beijing 100871, China

*Correspondence: Yong Nie, Nieyong@pku.edu.cn, and Xiao-Lei Wu

[xiaolei_wu@pku.edu.cn](mailto:xiaolei_wu@pku.edu.cn)

Tel: +86 10-62759047; Fax: +86 10-62759047

Running title: Heme recycling and sharing by mycomembrane vesicles

**Materials and Methods**

**Microscopy**

The whole mount immunostaining was used to identify the location of *Dt*HtaA in membrane vesicles ([1](#_ENREF_1)). Briefly, the membrane vesicles were absorbed to the EM grids for 5 minutes, and the grids were blocked with PBS-BSA for 30 minutes. Then, the grids were incubated with rabbit polyclonal antibody anti-*Dt*HtaA for 1 hour. After incubation with ImmunoGold-labeled anti-rabbit IgG, grids were observed at 80 kV by Hitachi HT7700. Fluorescence microscope (Leica DM6000) was used to identify whether the fluorescence labelled mMVs fused to recipient cells. The FM4-64 labelled mMVs were incubated with recipient cells for 1 hour. The incubated cells were then washed 3 times (8000 rpm, 2 min) with PBS buffer to remove the free mMVs. The cells from different species were incubated with FM4-64 (3.3 μg/mL in PBS buffer at 37°C for 10 min) to testify the label efficiency of FM4-64. For observation, the excitation wavelength were 520-600 nm and the emission wavelength were 570-720 nm.

**Assessment of membrane vesicles secretion**

To test whether DQ12-45-1b actively released membrane vesicles, membrane vesicles from live cells and dead cells were collected. Briefly, DQ12-45-1b (OD_600_=0.1) was incubated in iron-limited condition for 36 h to the mid-exponential phase (OD_600_~1.7). Then all the cells were collected by centrifugation (8000 rpm, 5 min), and washed by PBS solution twice. Half of the cells were incubated at 80°C for 2 hours to achieve heat-kill cells. All the heat-killed cells and live cells were re-inoculated into minimal medium (defined minimal medium amended with 0.5% sodium acetate) for cultivation (OD_600_~1.7). The cultures were collected after incubated at 30°C for 72 h, and the membrane vesicles were harvested as described before.

**Membrane fraction separation**

To identify the subcellular location of *Dt*HtaA in DQ12-45-1b, the inner and outer membrane was separated as previously described ([2](#_ENREF_2)). Briefly, DQ12-45-1b cells were harvested from 1-liter iron-limited medium at late-exponential phase by centrifugation (8000 rpm, 15 min). Cells were then washed twice using PBS buffer and broken by a High-Pressure Breaking method (Juneng, Guangzhou, China) ([3](#_ENREF_3)). The leaving intact cells were removed by centrifugation at 4 000 ×g for 30 min. Supernatants were collected and for further ultracentrifugation at 150 000 ×g for 1 hour. The pellets containing membrane fraction and the supernatants containing cytoplasm were collected, respectively. Then the membrane fraction was purified by sucrose density gradient centrifugation (56% and 20% (*w/v*), 150 000 ×g, 2 h). The bands between 56% and 20% sucrose were collected and layered on a sucrose gradient (56%, 53%, 50%, 47%, 44%, 41%, 38%, and 35%) in SW55Ti 5 mL tubes. After centrifugation at 200 000 ×g for 42 h, two bands were detected and collected. The protein concentration was measured by BCA method, and the NADH oxidase activity was determined to identify the inner membrane fraction ([2](#_ENREF_2)). Briefly, samples were diluted in 100 mM Tris-HCl (pH 7.5) containing 0.25 mM NADH, 5 mM MgCl_2_, and 10 mM CaCl_2_. The consumption of NADH was measured at 340 nm for up to 1 hour.

**Western blotting**

The protein samples from the membrane factions and mMVs were prepared in 2×SDS loading buffer ([4](#_ENREF_4)). After boiling for 10 minutes, samples with same protein contents were analyzed by SDS-PAGE, followed by semi-dry transfer to PVDF membrane (100 mA, 30 min). Then the membrane was blocked by 5% BSA at room temperature for 1 hour. The membrane was then washed and incubated with anti-*Dt*HtaA (Pujian biotechnology, Wuhan, China, diluted 1/1500 in TBST) for 2 hours, followed by washing in TBST for 3 times. For signal detection, the membrane was incubated with anti-rabbit-HRP secondary antibody. The visualization was conducted using DAB reagent kit (Tiangen, Beijing, China).

**Table S1.** Primers used in this research

| Primers | Sequence (5’-3’) |
| --- | --- |
| DthtaA_LF1 | GGACCACGACGACCGTGAC |
| DthtaA_LF2 | CGAGCAGGTCTACGTGGTCCT |
| DthtaA_LR1 | CCTTCATCCGTTTCCACGGTGGCCCGATAACTGGTCACTG |
| DthtaA_RF1 | TTAAGCGTGCATAATAAGCCCTAACCTGTTCGGCAGCCTGAT |
| DthtaA_RR1 | CGGAGAACGCCTTGTTCATC |
| DthtaA_RR2 | CCGAGACTGTCCGCCATTCT |
| DthtaA_idtF | AGGTCACCTGCGGTTTCCTC |
| DthtaA_idtR | ACAAGCCCGGAGGACTCATG |
| DthmuUV_LF1 | GATCGTCGAGACCGAACGAT |
| DthmuUV_LF2 | AGCCGGACGTCATCATCAT |
| DthmuUV_LR1 | CCTTCATCCGTTTCCACGGTGACGCTCCATAACGTGGCAT |
| DthmuUV_RF1 | TTAAGCGTGCATAATAAGCCCTTCCGAACTGGTGGAAGAGGT |
| DthmuUV_RR1 | AGGTGAGGAAACCGCAGGT |
| DthmuUV_RR2 | GTCCGGGATGTAGTCGTAGGT |
| DthmuUV_idtF | GGATCCCTGTCATCGTGGTG |
| DthmuUV_idtR | GGGTGAAGGTCTCGTTGGC |
| P18F | CGGCCGCGACTCTACAATT |
| P18R | GGTGGCGACCAGCTCTTGT |
| DthtaA_F | TTGAACAAGAGCTGGTCGCCACCATGACCAGTGACCAGTTA |
| DthtaA_R | AATTGTAGAGTCGCGGCCGTCAGTCCTTTCGTCGAACT |
| DthmuUV_F | TTGAACAAGAGCTGGTCGCCACCATGGTGACCGGGGGCCTGCT |
| DthmuUV_R | AATTGTAGAGTCGCGGCCGTCACGAGAACTCCTTGAC |

Figure S1. DQ12-45-1b actively released membrane vesicles. After incubated at 30°C for 72 h, the membrane vesicles were collected from heat-killed cells (left) and live cells (right). Only the live cells secret membrane vesicles.


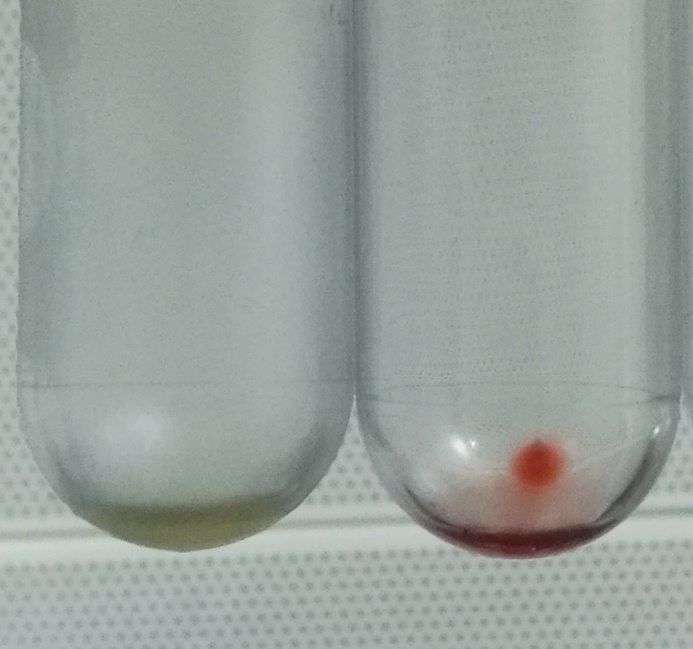


Figure S2. DQ12-45-1b was hypervesiculation at iron-limiting condition. A. TEM micrograph of DQ12-45-1b cells cultured in iron-rich medium at the late-exponential phase. B. TEM micrograph of DQ12-45-1b cells cultured in iron-limiting medium at the late-exponential phase. C. Quantification of mMVs from iron-rich (FeCl_3_=40 μM) and iron-limiting (FeCl_3_=8 μM) medium. The mMVs were collected and incubated with 3.3 μg/mL FM4-64 at 37°C for 10 min, and the fluorescence intensity of both mMVs were identified by microplate reader with excitation at 509 nm and emission at 750 nm. Error bar represented three independent experiments.





Figure S3. Proteins with significant changes between mMVs from iron-limiting and iron-rich conditions. Changed proteins with *p*<0.05, and fold change > 1.5 or < 0.75 were analyzed. The corresponding thresholds were shown. NS, no significant changes. Up, up-regulated proteins in mMVs from iron-limiting condition. Down, down-regulated proteins in mMVs from iron-limiting condition.


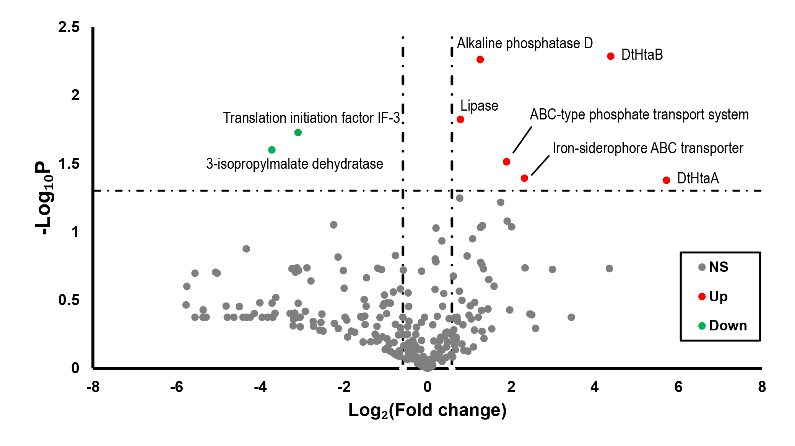


Figure S4. Conserved domains of HtaA (A) and gene arrangements (B) of HtaAB-HmuTUV from *Dietzia* sp. DQ12-45-1b and *Corynebacterium diphtheria*. SP, signal peptide.





Figure S5. AlkX exhibits no heme binding capacity. AlkX was incubated in 10 μM heme solution for 30 min. Excess heme was washed out and the protein was re-purified. The protein solution was scanned from 350-500 nm. AlkX without heme addition was used for comparison.





Figure S6. *Dt*HtaA achieves heme binding from varies hemoproteins. Purified *Dt*HtaA protein (5 μM) were incubated with 50 μM four hemoproteins: cytochrome P450, lignin peroxidase, cytochrome *c*, and catalase, respectively, at 30°C for 2 hours. Then, *Dt*HtaA were purified by the Ni affinity chromatography with the OD values detected from 350 nm to 500 nm.


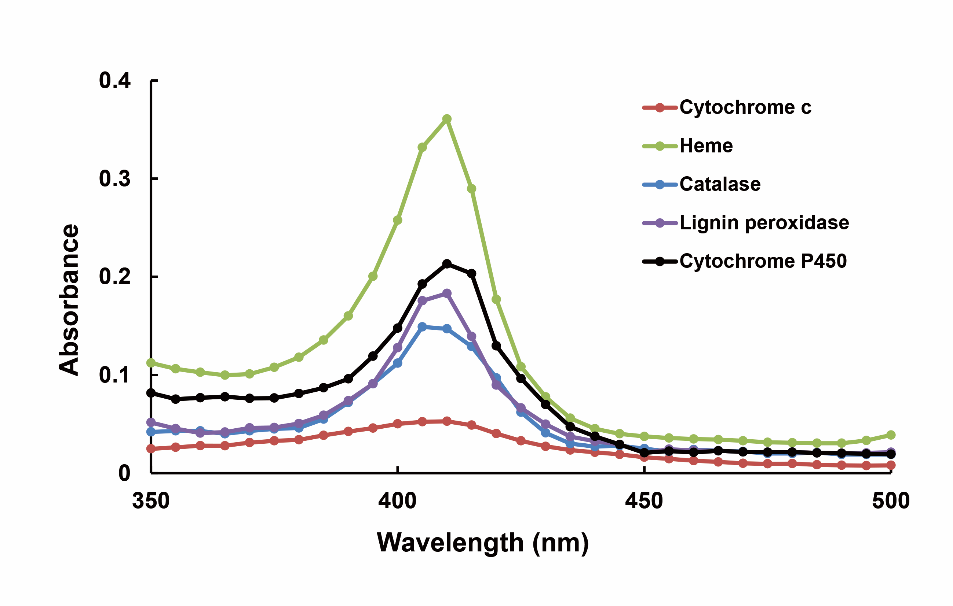


Figure S7. *Dt*HtaA was located on the surface of cells and mMVs. A. Membrane fraction separation by sucrose density gradient centrifugation. Cells were broken and membrane fractions were collected by ultracentrifugation. Further twice sucrose density gradient centrifugation was performed to form the upper yellow fraction (Y) and lower red fraction (R). B. NADH oxidase activity measured from Y fraction, R fraction, and cytoplasm. The cytoplasm was collected from the supernatant of ultracentrifugation of cell lysis solution. The reduction of NADH was determined at 340 nm for every 5 min. NC, negative control. The yellow fraction showed NADH oxidase activity, indicating this fraction was inner membrane. In addition, the red fraction with the same color of the mycomembrane vesicles (Figure S1) suggested that this faction was mycomembrane. C. Western blotting of *Dt*HtaA from cell fragments. The mMVs, red fraction, yellow fraction, and cyto (cytoplasm) were collected and separated by SDS-PAGE. Western blot was performed using anti-*Dt*HtaA. The band was only identified from mMVs and red fraction lane (mycomembrane) with similar signal intensity. Therefore, this result indicated that the increase of *Dt*HtaA abundance in iron-limiting mMVs reflects its enrichment in the mycomembrane. D. The whole mount immunostaining inspection of mMVs from iron-limited condition. mMVs were absorbed to EM grids, followed by BSA blocking, anti-*Dt*HtaA hybridization, and ImmunoGold-labeled anti-rabbit IgG incubation. The gold particles represented the location of *Dt*HtaA in mMVs. Bar = 100 nm.


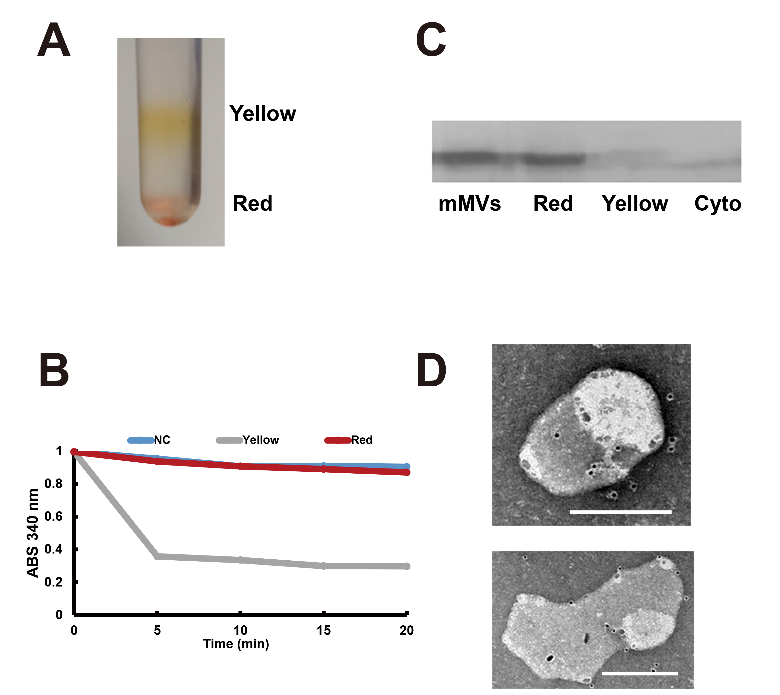


Figure S8. *Dt*HtaA in recipient cells is involved in utilization free heme and hemoproteins as sole iron source. DQ12-45-1b and Δ*DthtaA* strains were incubated in defined minimal medium supplemented with 0.5% sodium acetate (OD_600_=0.1). Cytochrome P450 (2 μM), lignin peroxidase (2 μM), cytochrome c (2 μM), and catalase (0.5 μM, four heme molecules in one catalase molecule) was added as sole iron source to the minimal medium, respectively. Heme, 2 μM heme was added to medium as sole iron source; NC, no heme added to medium. The strains were incubated and the maximal OD_600_ were recorded. The maximal growth of DQ12-45-1b in “Heme” was calibrated as 100%. Error bar represented three independent experiments. Knockout of *DthtaA* significantly impaired utilization of cytochrome P450, catalase, and lignin peroxidase for growth. However, there were still significant growth after knockout of *DthtaA* when compared with negative control, indicating that there may be some nonspecific proteins involved in heme assimilation from hemoproteins. These results suggested that *Dt*HtaA in recipient cells dominates the utilization of both free heme molecules and hemoproteins to support cell growth.





Figure S9. *Dt*HmuUV is not involved in mMV recruit or membrane fusion. mMVs from DQ12-45-1b were FM4-64 labelled and incubated with DQ12-45-1b and Δ*DthmuUV* cells. After incubation for 1 hour, cells were collected and washed by PBS. A, phase contrast field of Δ*DthmuUV* cells; B, fluorescence field of Δ*DthmuUV* cells; C, phase contrast field of DQ12-45-1b cells; D, fluorescence field of DQ12-45-12b cells. The exposure time of all fluorescence field pictures was 100 ms. Bar = 5 μm. After incubation fluorescence labelled mMVs with DQ12-45-1b and Δ*DthmuUV* strains, we observed that both strains were labelled, with no significant difference in fusion efficiencies and fluorescence intensities (measured by microplate reader). Therefore, we conclude that *Dt*HmuUV participates in both mMV-based heme and free heme delivery, but is not essential in membrane fusion during mMV recruit.


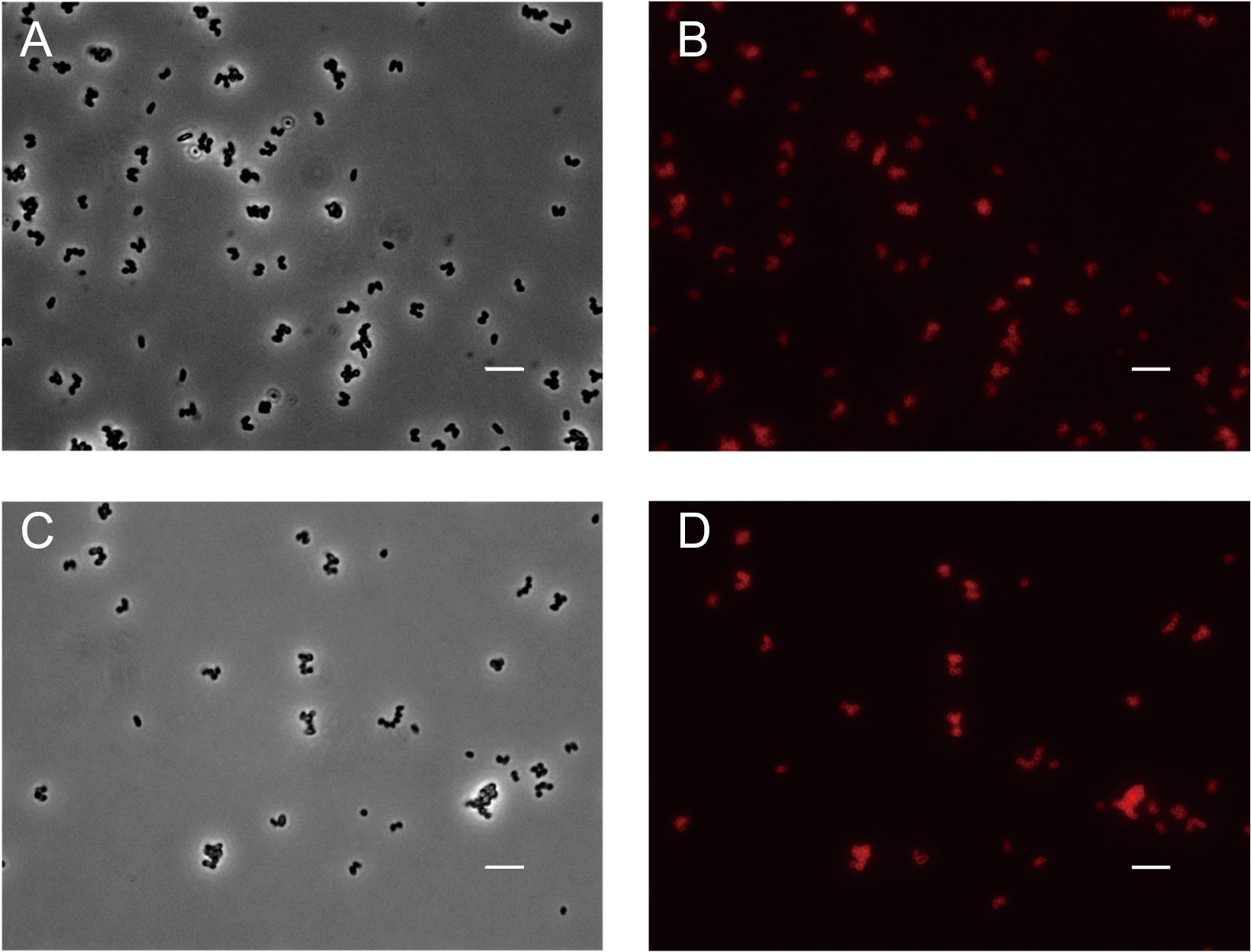


Figure S10. Microscopic images of recipient cells after incubated with FM4-64 labelled mMVs. A and C, phase contrast field of different cells; B, fluorescence field of recipient cells after incubated with FM4-64 labelled mMVs; D, fluorescence field of cells incubated with FM4-64. The exposure time of all fluorescence field pictures was 100 ms. Arrows indicated some weak labelled cells. Bar = 5 μm. The quantitative analysis of fluorescence intensity was shown in Figure 4A in the main text. After incubation cells with FM4-64, all the cells were labelled (C&D) with similar fluorescence intensity. After incubation with FM4-64 labelled mMVs, the recipient cells from *Dietzia* were all labelled with fluorescent signals, although the signal intensity was weaker than that of DQ12-45-1b. About 83% and 75% recipient cells from *C*. *glutamicum* and *R*. *erythropolis* were labelled after incubation with FM4-64 labelled mMVs, respectively, indicating the lower fusion efficiency compared with *Dietzia* cells. When we used *M*. *smegmatis*, *P*. *fluorescens*, *E*. *coli*, *B*. *subtilis*, *B*. *cereus*, and *A*. *baumannii* as recipient cells, cells labelled with fluorescent signals were much fewer than *Dietzia* cells. These results reassured our conclusion from Figure 4A that the fluorescent signals of recipient cells reflected the fusion efficiency. The microscopic images agree with the quantitative analysis of cell fluorescence intensities in Figure 4A.


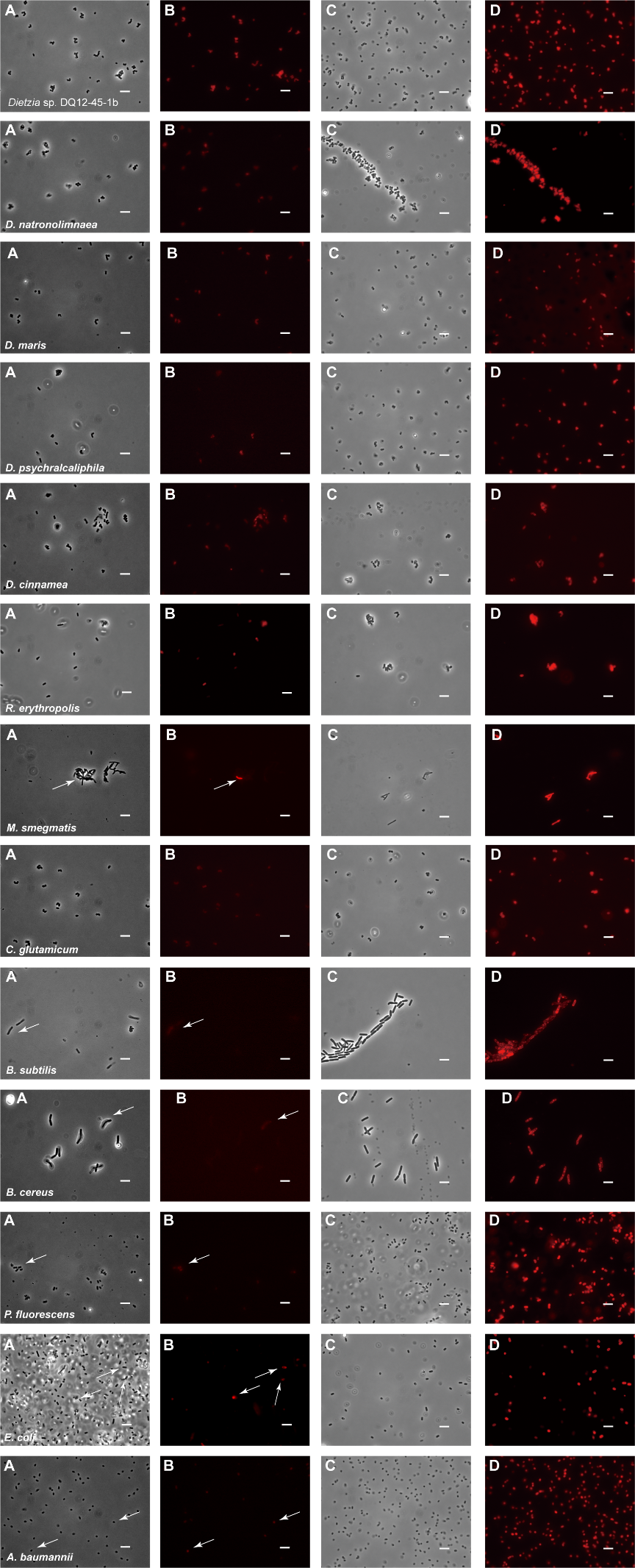


Figure S11. Correlation analysis of fusion efficiency and heme delivery efficiency. The fusion efficiency and heme delivery efficiency were identified in Figure 4A.





**References**

1. **Jung MK, Mun JY.** 2018. Sample preparation and imaging of exosomes by transmission electron microscopy. Journal of Visualized Experiments **131:**e56482.

2. **Marchand CH, Salmeron C, Bou Raad R, Meniche X, Chami M, Masi M, Blanot D, Daffe M, Tropis M, Huc E, Le Marechal P, Decottignies P, Bayan N.** 2012. Biochemical disclosure of the mycolate outer membrane of *Corynebacterium glutamicum*. J Bacteriol **194:**587-597.

3. **Liang JL, Nie Y, Wang M, Xiong G, Wang YP, Maser E, Wu XL.** 2016. Regulation of alkane degradation pathway by a TetR family repressor via an autoregulation positive feedback mechanism in a Gram‐positive *Dietzia* bacterium. Molecular microbiology **99:**338-359.

4. **Sambrook J, Fritsch EF, Maniatis T.** 1989. Molecular cloning: a laboratory manual. Cold spring harbor laboratory press.
