# Supplementary figures and images for: Extracellular heme recycling and sharing across species by novel mycomembrane vesicles of a Gram-positive bacterium
Source: ISME J. 2020 Oct 9;15(2):605–17. doi: 10.1038/s41396-020-00800-1 (PMC8027190; doi:10.1038/s41396-020-00800-1)

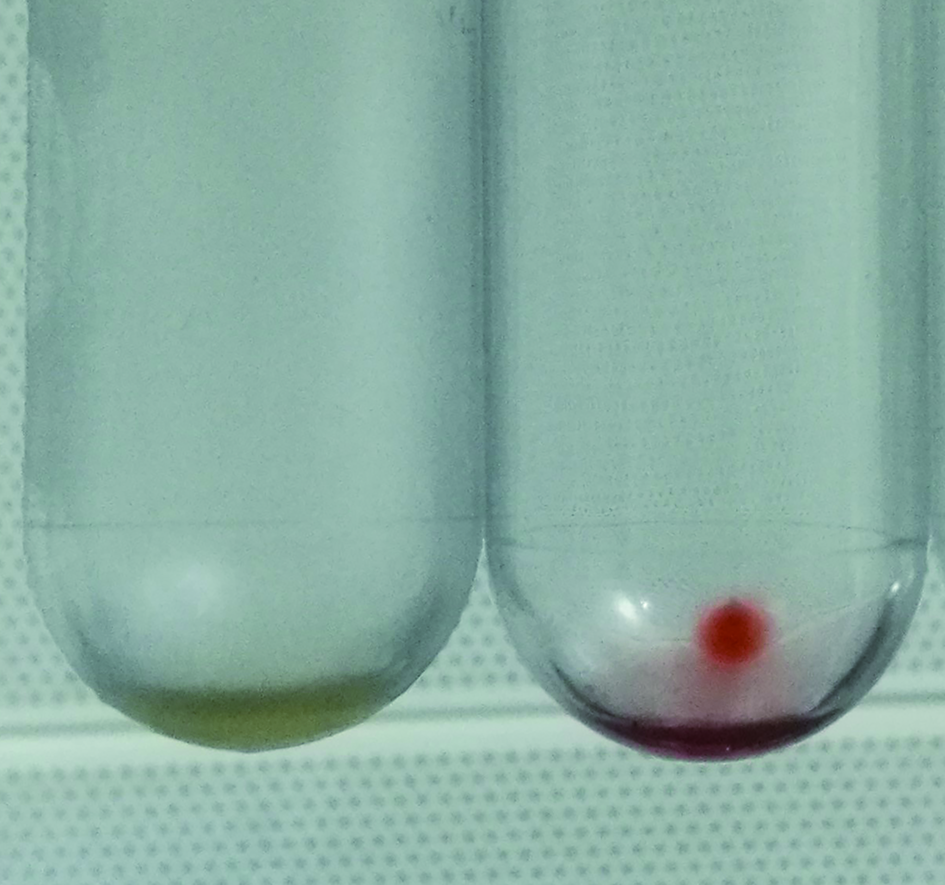

Supplement: Supplementary file 2 — Figure S1 [file 41396_2020_800_MOESM2_ESM.tif]

**A**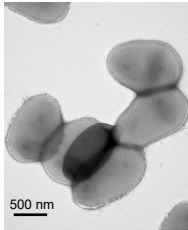**B**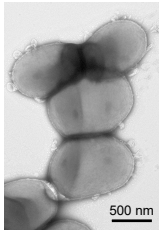**C**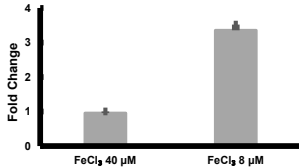

Supplement: Supplementary file 3 — Figure S2 [file 41396_2020_800_MOESM3_ESM.pdf]

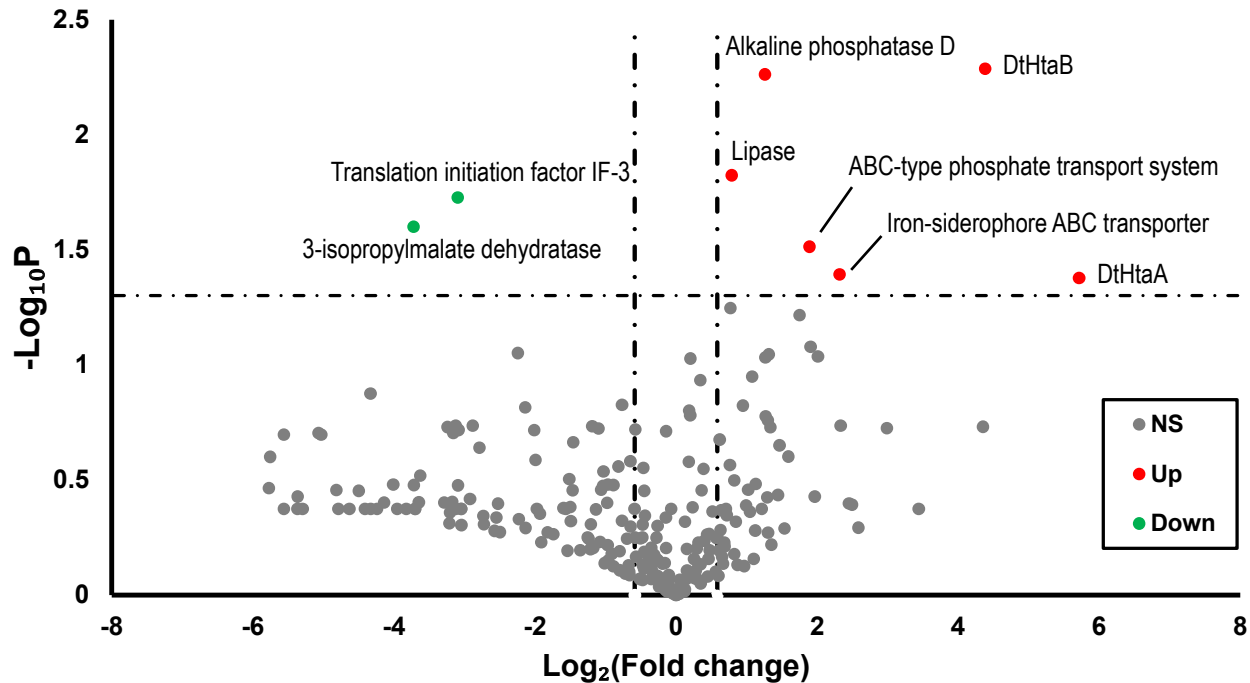

Supplement: Supplementary file 4 — Figure S3 [file 41396_2020_800_MOESM4_ESM.pdf]

**A**

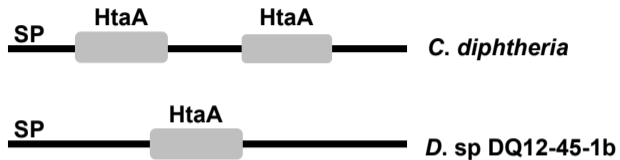

**B**

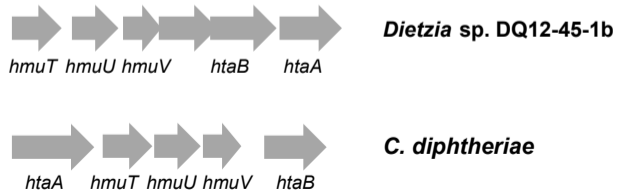

Supplement: Supplementary file 5 — Figure S4 [file 41396_2020_800_MOESM5_ESM.pdf]

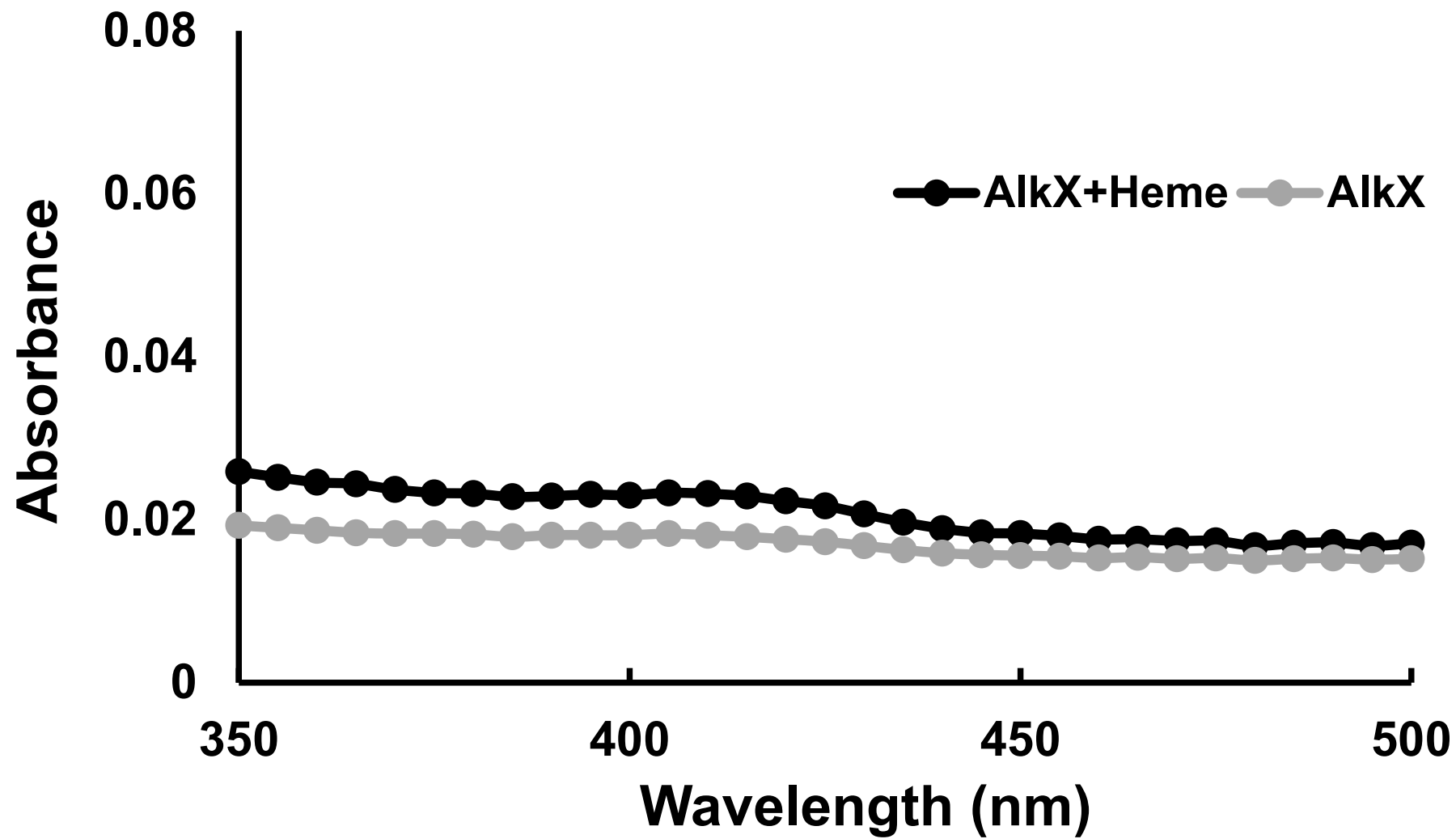

Supplement: Supplementary file 6 — Figure S5 [file 41396_2020_800_MOESM6_ESM.pdf]

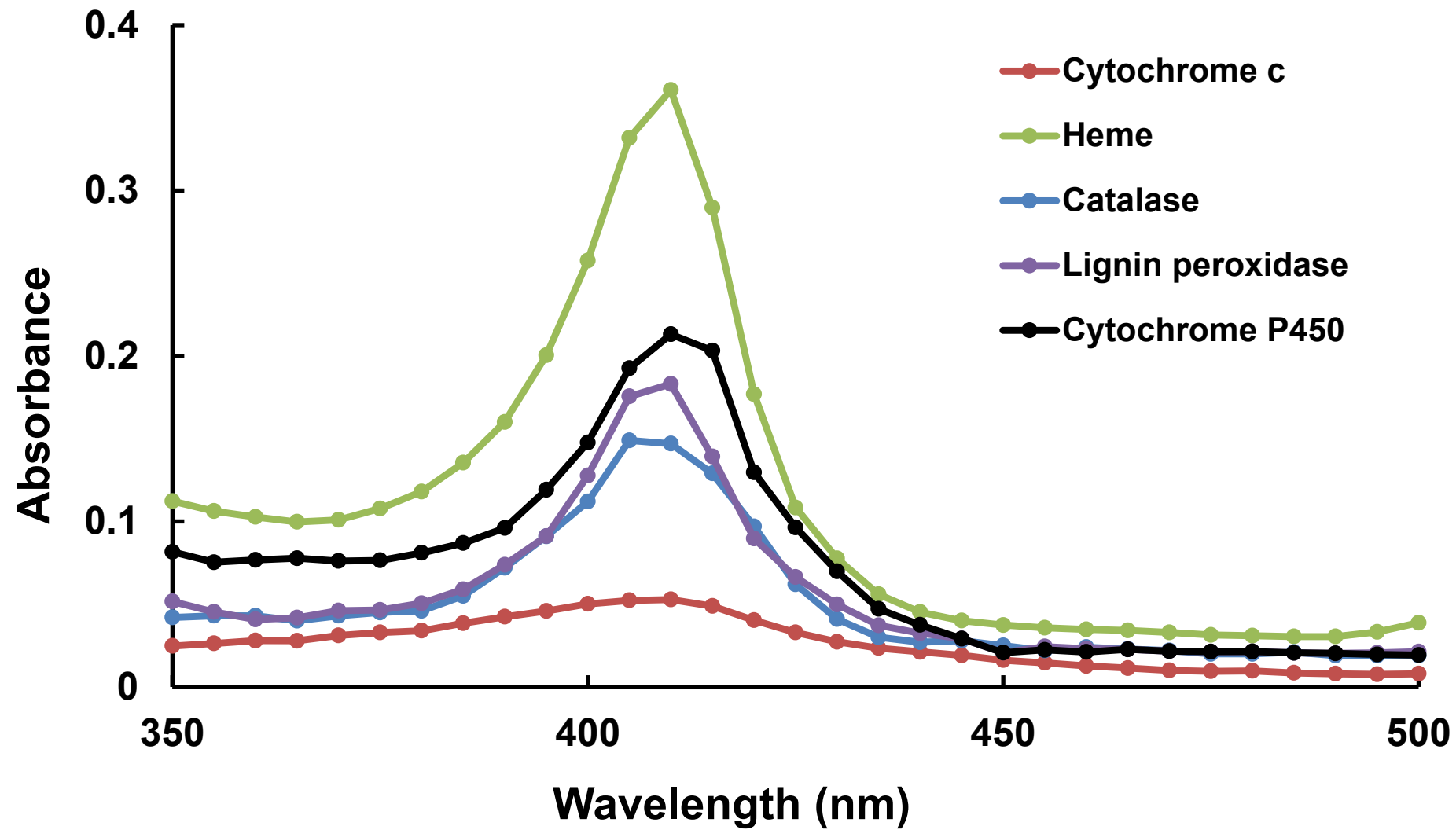

Supplement: Supplementary file 7 — Figure S6 [file 41396_2020_800_MOESM7_ESM.pdf]

**A**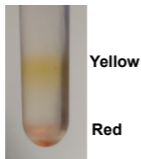**C**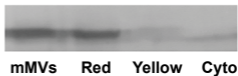**B**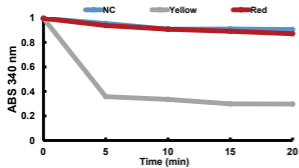**D**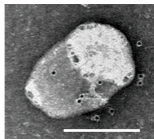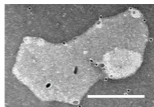

Supplement: Supplementary file 8 — Figure S7 [file 41396_2020_800_MOESM8_ESM.pdf]

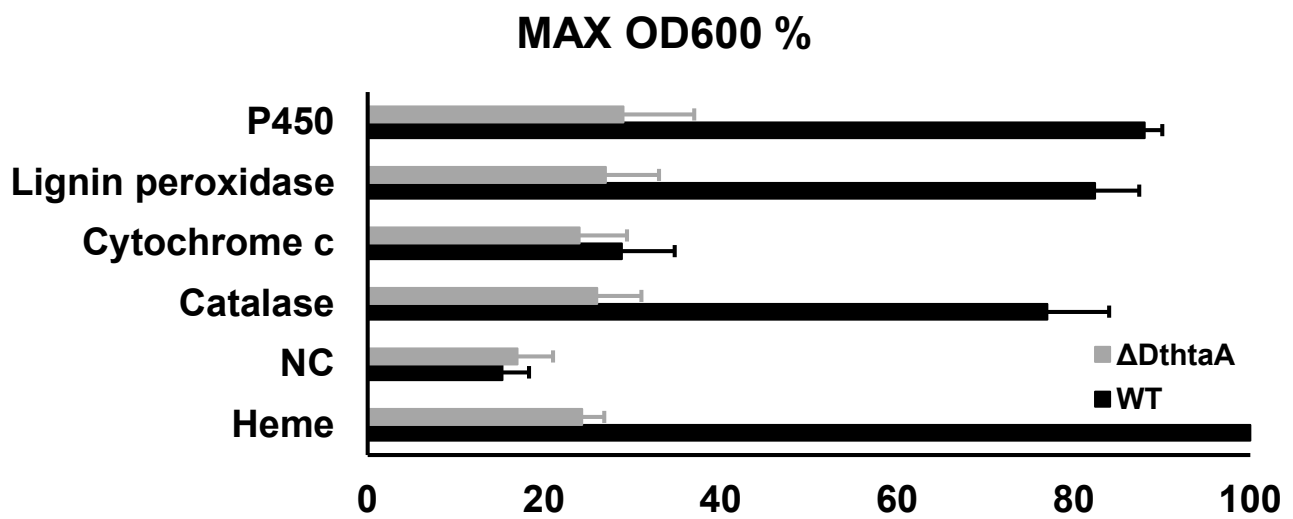

Supplement: Supplementary file 9 — Figure S8 [file 41396_2020_800_MOESM9_ESM.pdf]

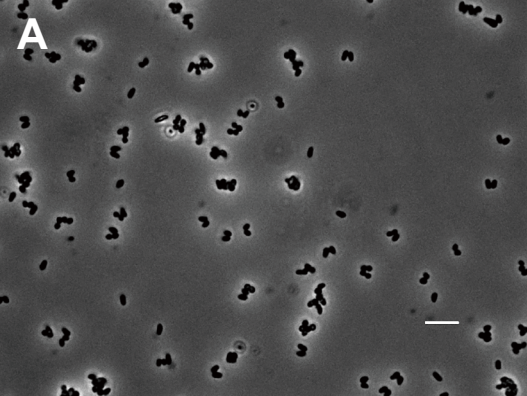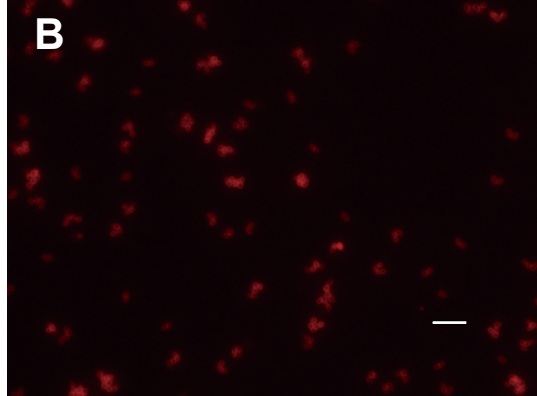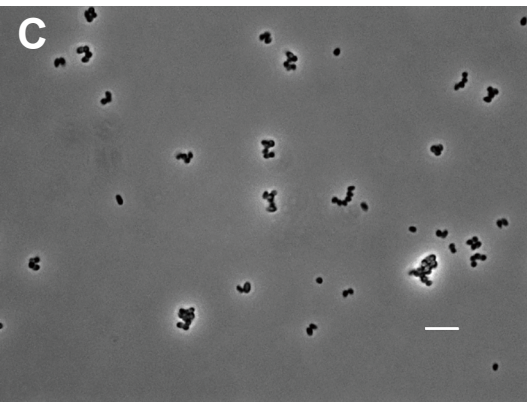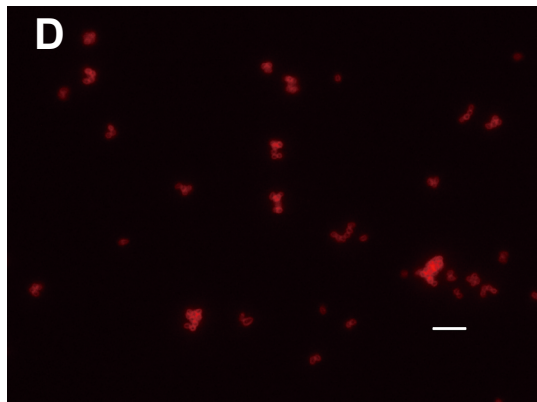

Supplement: Supplementary file 10 — Figure S9 [file 41396_2020_800_MOESM10_ESM.pdf]

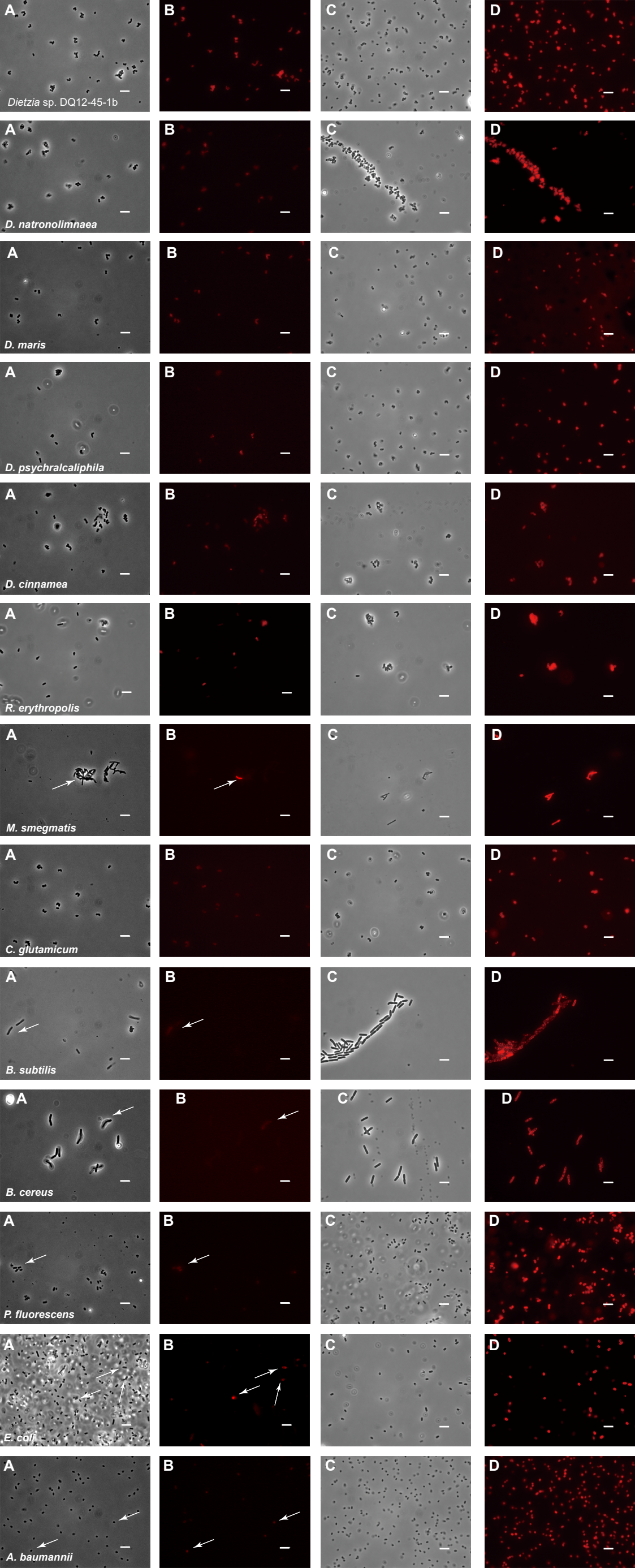

Supplement: Supplementary file 11 — Figure S10 [file 41396_2020_800_MOESM11_ESM.pdf]

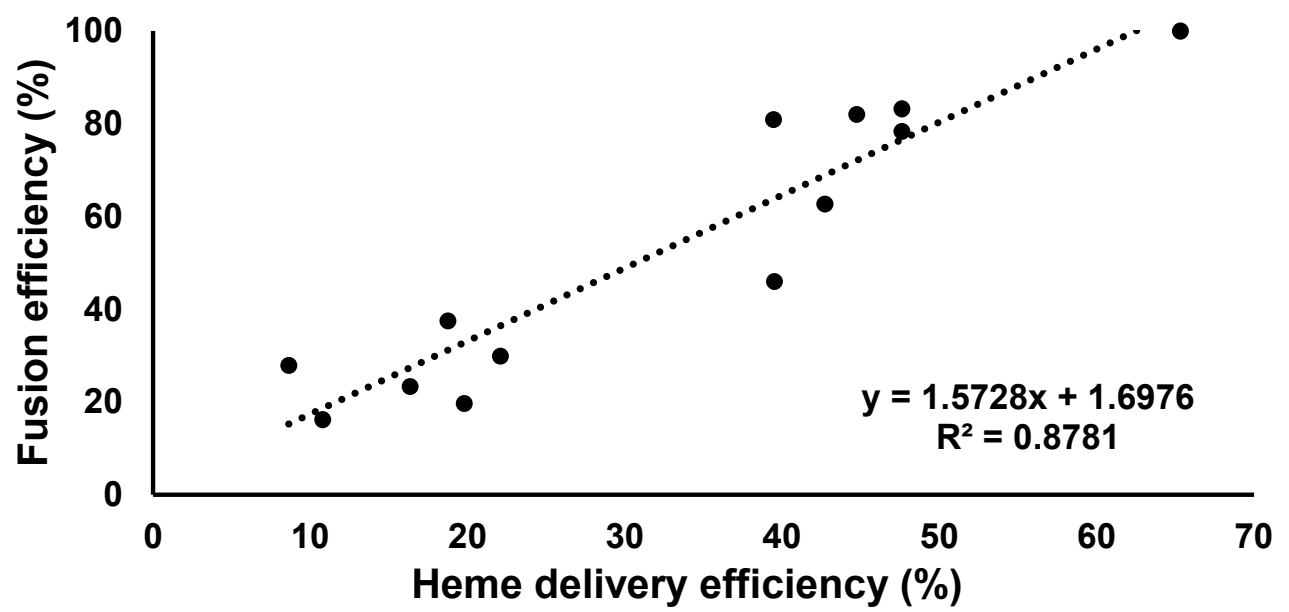

Supplement: Supplementary file 12 — Figure S11 [file 41396_2020_800_MOESM12_ESM.pdf]
