# Supplementary material for: Extracellular heme recycling and sharing across species by novel mycomembrane vesicles of a Gram-positive bacterium
Source: ISME J. 2020 Oct 9;15(2):605–17. doi: 10.1038/s41396-020-00800-1 (PMC8027190; doi:10.1038/s41396-020-00800-1)
Supplement: Supplementary file 14 — Table S2 (pdf version) [file 41396_2020_800_MOESM14_ESM.pdf]

Table S2
Proteomes of H-mMVs (iron-rich conditions) and L-mMVs (iron-limiting conditions) determined by LC-MS/MS
The peptide counts based relative protein abundance was shown.
The proteins peptide fragments were collected and searched against the DQ12-45-1b genome database (GCA_009740915.1).
The subcellular location prediction was performed as described in methods.
The annotation of proteins was conducted against NCBI protein NR database.

Contig	L-mMVs-1	L-mMVs-2	L-mMVs-3	Location	Protein ID	Annotation
Contig46-orf04635	0.126	0.089	0.079	Unknown	QGW26039.1	FIG00995049: hypothetical protein
Contig46-orf01248	0.060	0.037	0.029	mycomembrane	QGW23845.1	hypothetical protein
Contig46-orf00469	0.041	0.129	0.083	mycomembrane	QGW23367.1	putative esterase
Contig46-orf04414	0.039	0.058	0.060	mycomembrane	QGW25899.1	putative esterase
Contig46-orf05111	0.037	0.029	0.016	mycomembrane	QGW26366.1	Triacylglycerol lipase precursor (EC 3.1.1.3)
Contig46-orf03860	0.032	0.010	0.004	Cytoplasmic	QGW25535.1	DNA-directed RNA polymerase beta' subunit (EC 2.7.7.6)
Contig46-orf05236	0.028	0.032	0.045	mycomembrane	QGW26449.1	hypothetical protein
Contig46-orf04551	0.027	0.014	0.030	mycomembrane	QGW25988.1	No significant database matches
Contig46-orf01781	0.027	0.000	0.001	Cytoplasmic	QGW24190.1	Ribonuclease E (EC 3.1.26.12)
Contig46-orf03771	0.025	0.021	0.047	Extracellular	QGW25474.1	putative esterase
Contig46-orf04633	0.022	0.012	0.002	Cytoplasmic	QGW26037.1	3-oxoacyl-[acyl-carrier protein] reductase (EC 1.1.1.100)
Contig46-orf01151	0.020	0.014	0.014	mycomembrane	QGW23786.1	arabinan endo-1,5-alpha-L-arabinosidase A precursor
Contig46-orf05343	0.020	0.010	0.009	Periplasmic	QGW26515.1	Maltose/maltodextrin ABC transporter, substrate binding periplasmic protein MalE
Contig46-orf05062	0.020	0.029	0.022	mycomembrane	QGW26334.1	Antigen 85-B precursor (85B)
Contig46-orf04151	0.017	0.008	0.012	mycomembrane	QGW25732.1	predicted hydrolase or acyltransferase
Contig46-orf04700	0.016	0.022	0.022	mycomembrane	QGW26085.1	putative lipoprotein
Contig46-orf00456	0.015	0.021	0.027	mycomembrane	QGW23358.1	N-acetylmuramoyl-L-alanine amidase
Contig46-orf05031	0.013	0.003	0.001	Unknown	QGW26312.1	Glucose-methanol-choline (GMC) oxidoreductase:NAD binding site
Contig46-orf02292	0.012	0.006	0.010	mycomembrane	QGW24525.1	hypothetical protein
Contig46-orf00470	0.012	0.024	0.026	mycomembrane	QGW23368.1	putative esterase
Contig46-orf04534	0.012	0.012	0.007	Extracellular	QGW25976.1	secreted alkaline phosphatase
Contig46-orf01247	0.010	0.010	0.008	Periplasmic	QGW23844.1	hypothetical protein
Contig46-orf02149	0.010	0.008	0.011	Periplasmic	QGW24429.1	periplasmic binding protein
Contig46-orf03780	0.010	0.001	0.005	Cytoplasmic	QGW25482.1	LSU ribosomal protein L2p (L8e)
Contig46-orf05270	0.010	0.001	0.003	CytoplasmicMembrane	QGW26474.1	Multimodular transpeptidase-transglycosylase (EC 2.4.1.129) (EC 3.4.-.-)
Contig46-orf02296	0.009	0.006	0.006	mycomembrane	QGW24526.1	hypothetical protein
Contig46-orf04907	0.009	0.013	0.015	mycomembrane	QGW26230.1	Putative secreted protein
Contig46-orf03776	0.008	0.000	0.011	Cytoplasmic	QGW25479.1	SSU ribosomal protein S3p (S3e)
Contig46-orf03658	0.008	0.003	0.026	mycomembrane	QGW25397.1	Phage peptidoglycan binding endopeptidase
Contig46-orf00641	0.008	0.006	0.019	mycomembrane	QGW23470.1	Putative uncharacterized protein BCG_3873
Contig46-orf04606	0.008	0.003	0.011	mycomembrane	QGW26021.1	Putative serine protease
Contig46-orf00508	0.008	0.006	0.000	CytoplasmicMembrane	QGW23387.1	Alkane-1 monooxygenase (EC 1.14.15.3)
Contig46-orf04818	0.007	0.007	0.005	mycomembrane	QGW26166.1	FIG00547498: hypothetical protein
Contig46-orf00784	0.007	0.001	0.029	mycomembrane	QGW23561.1	Multimodular transpeptidase-transglycosylase (EC 2.4.1.129) (EC 3.4.-.-)
Contig46-orf00728	0.006	0.005	0.003	mycomembrane	QGW23531.1	Allergen V5/Tpx-1 family protein
Contig46-orf04699	0.006	0.009	0.015	mycomembrane	QGW26084.1	possible conserved lipoprotein
Contig46-orf02524	0.005	0.009	0.006	mycomembrane	QGW24679.1	hypothetical protein
Contig46-orf01647	0.005	0.001	0.002	CytoplasmicMembrane	QGW24096.1	[Acyl-carrier-protein] acetyl transferase of FASI (EC 2.3.1.38)
Contig46-orf04548	0.005	0.006	0.008	mycomembrane	QGW25985.1	hypothetical protein
Contig46-orf03066	0.005	0.003	0.002	Cytoplasmic	QGW25011.1	ATP synthase alpha chain (EC 3.6.3.14)
Contig46-orf04646	0.005	0.004	0.000	Unknown	QGW26046.1	Copper metallochaperone, bacterial analog of Cox17 protein
Contig46-orf05129	0.005	0.003	0.003	mycomembrane	QGW26377.1	BNR repeat domain protein
Contig46-orf01420	0.005	0.001	0.001	CytoplasmicMembrane	QGW23947.1	Succinate dehydrogenase flavoprotein subunit (EC 1.3.99.1)
Contig46-orf01212	0.005	0.005	0.003	Periplasmic	QGW23825.1	Phosphate ABC transporter, periplasmic phosphate-binding protein PstS (TC 3.A.1.7.1)
Contig46-orf02580	0.005	0.003	0.012	Cytoplasmic	QGW24711.1	hypothetical protein
Contig46-orf04405	0.005	0.006	0.005	CytoplasmicMembrane	QGW25894.1	hypothetical protein
Contig46-orf00032	0.004	0.003	0.000	CytoplasmicMembrane	QGW23091.1	Alkane-1 monooxygenase (EC 1.14.15.3)
Contig46-orf02028	0.004	0.002	0.013	mycomembrane	QGW24350.1	putative secreted protein
Contig46-orf02007	0.004	0.001	0.002	CytoplasmicMembrane	QGW24337.1	Cytochrome c oxidase polypeptide II (EC 1.9.3.1)
Contig46-orf02017	0.004	0.001	0.001	CytoplasmicMembrane	QGW24343.1	Ubiquinol--cytochrome c reductase, cytochrome B subunit (EC 1.10.2.2)
Contig46-orf04522	0.004	0.003	0.004	mycomembrane	QGW25968.1	hypothetical protein

Contig46-orf03014	0.004	0.003	0.005	mycomembrane	QGW24980.1	possible glucose dehydrogenase
Contig46-orf01366	0.004	0.003	0.003	Unknown	QGW23915.1	No significant database matches
Contig46-orf01442	0.004	0.003	0.002	Periplasmic	QGW23962.1	Ferric iron ABC transporter, iron-binding protein
Contig46-orf04550	0.004	0.003	0.006	mycomembrane	QGW25987.1	hypothetical protein
Contig46-orf02892	0.004	0.001	0.001	Cytoplasmic	QGW24905.1	SSU ribosomal protein S2p (SAe)
Contig46-orf04386	0.004	0.000	0.000	Extracellular	QGW25882.1	DNA-binding protein HU / low-complexity, AKP-rich domain
Contig46-orf00729	0.004	0.001	0.003	mycomembrane	QGW23532.1	hypothetical protein
Contig46-orf04582	0.004	0.011	0.003	mycomembrane	QGW26008.1	putative serine/threonine protein kinase (putative secreted protein)
Contig46-orf02019	0.003	0.001	0.001	CytoplasmicMembrane	QGW24344.1	Ubiquinol-cytochrome C reductase iron-sulfur subunit (EC 1.10.2.2)
Contig46-orf00484	0.003	0.000	0.000	CytoplasmicMembrane	QGW23374.1	Probable arabinosyltransferase A (EC 2.4.2.-)
Contig46-orf02020	0.003	0.001	0.002	CytoplasmicMembrane	QGW24345.1	ubiquinol cytochrome C oxidoreductase, cytochrome C1 subunit
Contig46-orf04887	0.003	0.004	0.019	mycomembrane	QGW26217.1	hypothetical protein
Contig46-orf00500	0.003	0.000	0.000	Unknown	QGW23382.1	putative glycosyltransferase
Contig46-orf04812	0.003	0.001	0.001	CytoplasmicMembrane	QGW26163.1	Putative stomatin/prohibitin-family membrane protease subunit YbbK
Contig46-orf01484	0.003	0.000	0.001	Unknown	QGW23993.1	LpqB
Contig46-orf04713	0.003	0.002	0.002	mycomembrane	QGW26095.1	Lyzozyme M1 (1,4-beta-N-acetylmuramidase) (EC 3.2.1.17)
Contig46-orf04204	0.003	0.007	0.004	Extracellular	QGW25766.1	hypothetical protein
Contig46-orf00647	0.003	0.005	0.008	mycomembrane	QGW23474.1	Trypsin delta/gamma precursor (EC 3.4.21.4)
Contig46-orf04604	0.003	0.005	0.003	mycomembrane	QGW26020.1	putative protease
Contig46-orf01284	0.003	0.005	0.003	Unknown	QGW23866.1	glutamine cyclotransferase
Contig46-orf04864	0.003	0.000	0.000	Cytoplasmic	QGW26198.1	Cyclopropane-fatty-acyl-phospholipid synthase (EC 2.1.1.79)
Contig46-orf03752	0.003	0.005	0.012	mycomembrane	QGW25460.1	probable lipase
Contig46-orf03062	0.002	0.005	0.003	CytoplasmicMembrane	QGW25009.1	ATP synthase beta chain (EC 3.6.3.14)
Contig46-orf04365	0.002	0.002	0.001	mycomembrane	QGW25868.1	secreted lipase
Contig46-orf04608	0.002	0.000	0.000	CytoplasmicMembrane	QGW26022.1	Iron-sulphur-binding reductase
Contig46-orf04943	0.002	0.006	0.002	Periplasmic	QGW26255.1	glutamate-binding protein of ABC transporter system
Contig46-orf04415	0.002	0.001	0.018	Unknown	QGW25900.1	hypothetical protein
Contig46-orf02225	0.002	0.006	0.008	mycomembrane	QGW24478.1	Phage peptidoglycan binding endopeptidase
Contig46-orf03789	0.002	0.005	0.004	Cytoplasmic	QGW25488.1	Translation elongation factor Tu
Contig46-orf03129	0.002	0.002	0.000	Extracellular	QGW25054.1	hypothetical protein
Contig46-orf00832	0.002	0.000	0.000	Cytoplasmic	QGW23594.1	DNA topoisomerase I (EC 5.99.1.2)
Contig46-orf02266	0.002	0.004	0.002	Extracellular	QGW24503.1	hypothetical protein
Contig46-orf03775	0.002	0.000	0.001	Cytoplasmic	QGW25478.1	LSU ribosomal protein L16p (L10e)
Contig46-orf00406	0.002	0.001	0.002	Unknown	QGW23324.1	hypothetical protein
Contig46-orf03862	0.002	0.004	0.001	Cytoplasmic	QGW25536.1	DNA-directed RNA polymerase beta subunit (EC 2.7.7.6)
Contig46-orf00396	0.002	0.005	0.000	Cytoplasmic	QGW23315.1	Glutamate synthase [NADPH] large chain (EC 1.4.1.13)
Contig46-orf00538	0.002	0.001	0.001	mycomembrane	QGW23403.1	5'-nucleotidase (EC 3.1.3.5)
Contig46-orf02315	0.002	0.000	0.001	Cytoplasmic	QGW24539.1	LSU ribosomal protein L20p
Contig46-orf03107	0.002	0.000	0.001	Cytoplasmic	QGW25038.1	DEAD-box ATP-dependent RNA helicase CshA (EC 3.6.4.13)
Contig46-orf00095	0.001	0.002	0.001	mycomembrane	QGW23126.1	hypothetical protein
Contig46-orf01987	0.001	0.001	0.004	mycomembrane	QGW24326.1	L-asparaginase (EC 3.5.1.1)
Contig46-orf01276	0.001	0.001	0.003	Unknown	QGW23861.1	FIG00996461: hypothetical protein
Contig46-orf03759	0.001	0.000	0.002	Cytoplasmic	QGW25466.1	SSU ribosomal protein S5p (S2e)
Contig46-orf01876	0.001	0.002	0.001	mycomembrane	QGW24250.1	hypothetical protein
Contig46-orf00554	0.001	0.003	0.001	Unknown	QGW23411.1	hypothetical protein
Contig46-orf00742	0.001	0.001	0.001	Unknown	QGW23537.1	FIG00863414: hypothetical protein
Contig46-orf00363	0.001	0.004	0.002	CytoplasmicMembrane	QGW23293.1	Long-chain-fatty-acid--CoA ligase (EC 6.2.1.3)
Contig46-orf03348	0.001	0.000	0.001	Unknown	QGW25197.1	possible secreted protein
Contig46-orf01773	0.001	0.006	0.000	Cytoplasmic	QGW24186.1	Valyl-tRNA synthetase (EC 6.1.1.9)
Contig46-orf03063	0.001	0.001	0.000	Cytoplasmic	QGW25010.1	ATP synthase gamma chain (EC 3.6.3.14)
Contig46-orf01317	0.001	0.000	0.001	Cytoplasmic	QGW23884.1	LSU ribosomal protein L13p (L13Ae)
Contig46-orf05191	0.001	0.000	0.001	Periplasmic	QGW26418.1	Ferrous iron transport peroxidase EfeB

Contig46-orf00932	0.001	0.000	0.001	Cytoplasmic	QGW23656.1	ATP-dependent Clp protease, ATP-binding subunit ClpC
Contig46-orf05450	0.001	0.000	0.000	CytoplasmicMembrane	QGW26583.1	Inner membrane protein translocase component YidC, long form
Contig46-orf02032	0.001	0.000	0.000	Cytoplasmic	QGW24354.1	Long-chain fatty-acid-CoA ligase (EC 6.2.1.3), Mycobacterial subgroup FadD15
Contig8-orf00071	0.001	0.009	0.000	CytoplasmicMembrane	AFY63000.1	putative cytochrome P450 hydroxylase
Contig46-orf03003	0.001	0.001	0.001	Unknown	QGW24973.1	Ketol-acid reductoisomerase (EC 1.1.1.86)
Contig46-orf03792	0.001	0.004	0.000	Cytoplasmic	QGW25489.1	Translation elongation factor G
Contig46-orf00967	0.001	0.000	0.001	Periplasmic	QGW23677.1	Zinc ABC transporter, periplasmic-binding protein ZnuA
Contig46-orf02282	0.001	0.001	0.000	Periplasmic	QGW24518.1	ABC-type amino acid transport system, secreted component
Contig46-orf01657	0.001	0.000	0.001	mycomembrane	QGW24101.1	FIG00545237: hypothetical protein
Contig46-orf00609	0.001	0.000	0.001	mycomembrane	QGW23447.1	beta-lactamase domain protein
Contig46-orf01560	0.001	0.001	0.003	mycomembrane	QGW24039.1	hypothetical protein
Contig46-orf02995	0.001	0.003	0.001	Cytoplasmic	QGW24969.1	D-3-phosphoglycerate dehydrogenase (EC 1.1.1.95)
Contig3-orf00056	0.001	0.001	0.000	mycomembrane	NZ_CP046568.1	hypothetical protein
Contig46-orf00532	0.001	0.001	0.001	mycomembrane	QGW23400.1	Lipase 1 (EC 3.1.1.3)
Contig46-orf00412	0.001	0.000	0.000	Cytoplasmic	QGW23329.1	hypothetical protein
Contig46-orf03540	0.001	0.002	0.001	Cytoplasmic	QGW25326.1	Sphingolipid ceramide N-deacylase
Contig46-orf04628	0.001	0.003	0.000	Cytoplasmic	QGW26034.1	3-methylmercaptopropionyl-CoA dehydrogenase (DmdC)
Contig46-orf02583	0.001	0.003	0.003	Cytoplasmic	QGW24713.1	Aconitate hydratase (EC 4.2.1.3)
Contig46-orf00794	0.001	0.001	0.001	Cytoplasmic	QGW23569.1	cAMP-binding proteins
Contig46-orf00418	0.001	0.000	0.000	Cytoplasmic	QGW23333.1	3-hydroxyacyl-CoA dehydrogenase (EC 1.1.1.35)
Contig46-orf03746	0.001	0.001	0.001	Cytoplasmic	QGW25456.1	SSU ribosomal protein S4p (S9e)
Contig46-orf02097	0.001	0.001	0.000	Unknown	QGW24395.1	Putative secreted protein
Contig8-orf00074	0.001	0.004	0.000	Cytoplasmic	AFY63003.1	Ferredoxin reductase
Contig3-orf00065	0.001	0.001	0.000	mycomembrane	NZ_CP046568.1	putative involved in replication/partition
Contig46-orf03794	0.001	0.000	0.001	Cytoplasmic	QGW25491.1	SSU ribosomal protein S12p (S23e)
Contig46-orf03954	0.001	0.002	0.002	mycomembrane	QGW25595.1	hypothetical protein
Contig46-orf02810	0.001	0.000	0.000	Cytoplasmic	QGW24858.1	Phage shock protein A (IM30) , suppresses sigma54-dependent transcription
Contig46-orf02663	0.001	0.001	0.000	Cytoplasmic	QGW24765.1	S-adenosylmethionine synthetase (EC 2.5.1.6)
Contig46-orf02701	0.001	0.001	0.000	mycomembrane	QGW24786.1	FIG004453: protein YceG like
Contig46-orf02456	0.001	0.000	0.000	CytoplasmicMembrane	QGW24630.1	NADH dehydrogenase (EC 1.6.99.3)
Contig46-orf03793	0.000	0.000	0.001	Cytoplasmic	QGW25490.1	SSU ribosomal protein S7p (S5e)
Contig46-orf00035	0.000	0.001	0.001	Cytoplasmic	QGW23093.1	Oxidoreductase, short chain dehydrogenase/reductase family
Contig46-orf01868	0.000	0.000	0.000	Unknown	QGW24245.1	EpiH/GdmH-related protein
Contig46-orf02626	0.000	0.001	0.000	Cytoplasmic	QGW24742.1	Transketolase (EC 2.2.1.1)
Contig46-orf02849	0.000	0.001	0.000	Cytoplasmic	QGW24881.1	Translation initiation factor 2
Contig46-orf04726	0.000	0.004	0.000	Cytoplasmic	QGW26105.1	Phosphoenolpyruvate carboxykinase [GTP] (EC 4.1.1.32)
Contig46-orf00497	0.000	0.000	0.006	mycomembrane	QGW23380.1	probable triacylglycerol lipase(EC:3.1.1.3)
Contig3-orf00073	0.000	0.001	0.000	Periplasmic	NZ_CP046568.1	hypothetical protein
Contig46-orf02612	0.000	0.001	0.000	Unknown	QGW24732.1	Iron-sulfur cluster assembly protein SufB
Contig46-orf04397	0.000	0.003	0.000	Cytoplasmic	QGW25888.1	Isocitrate lyase (EC 4.1.3.1)
Contig46-orf05148	0.000	0.000	0.000	Cytoplasmic	QGW26389.1	Zinc ABC transporter, inner membrane permease protein ZnuB
Contig46-orf03071	0.000	0.000	0.000	CytoplasmicMembrane	QGW25013.1	ATP synthase F0 sector subunit b
Contig46-orf03340	0.000	0.001	0.000	mycomembrane	QGW25192.1	FIG01000940: hypothetical protein
Contig46-orf01954	0.000	0.000	0.001	Cytoplasmic	QGW24304.1	Glutamine synthetase type I (EC 6.3.1.2)
Contig46-orf04770	0.000	0.002	0.000	Cytoplasmic	QGW26134.1	Diaminobutyrate-pyruvate aminotransferase (EC 2.6.1.46)
Contig46-orf01419	0.000	0.000	0.000	CytoplasmicMembrane	QGW23946.1	Succinate dehydrogenase iron-sulfur protein (EC 1.3.99.1)
Contig46-orf01238	0.000	0.004	0.000	Cytoplasmic	QGW23839.1	Enoyl-CoA hydratase [isoleucine degradation] (EC 4.2.1.17)
Contig46-orf02030	0.000	0.001	0.003	Periplasmic	QGW24352.1	NLP/P60 family protein
Contig46-orf04839	0.000	0.001	0.002	mycomembrane	QGW26184.1	secreted lipoprotein, ErfK/YbiS/YcfS/YnhG family
Contig46-orf04994	0.000	0.001	0.007	Unknown	QGW26290.1	Triacylglycerol lipase precursor (EC 3.1.1.3)
Contig46-orf03909	0.000	0.001	0.001	Periplasmic	QGW25565.1	Tricarboxylate transport protein TctC
Contig46-orf05152	0.000	0.000	0.000	Unknown	QGW26391.1	Zinc ABC transporter, inner membrane permease protein ZnuB

Contig46-orf03157	0.000	0.002	0.000	Cytoplasmic	QGW25072.1	2,3,4,5-tetrahydropyridine-2,6-dicarboxylate N-succinyltransferase (EC 2.3.1.117)
Contig46-orf03959	0.000	0.000	0.003	Extracellular	QGW25598.1	LIPOPROTEIN VSAC (FRAGMENT)
Contig46-orf01589	0.000	0.003	0.000	Periplasmic	QGW24057.1	Beta-galactosidase (EC 3.2.1.23)
Contig46-orf00804	0.000	0.000	0.000	CytoplasmicMembrane	QGW23576.1	FIG00820727: hypothetical protein
Contig46-orf01545	0.000	0.003	0.000	Cytoplasmic	QGW24029.1	Collagen alpha 1(I) chain precursor
Contig46-orf03086	0.000	0.000	0.001	Cytoplasmic	QGW25022.1	Transcription termination factor Rho
Contig46-orf02637	0.000	0.000	0.000	Cytoplasmic	QGW24750.1	NAD-dependent glyceraldehyde-3-phosphate dehydrogenase (EC 1.2.1.12)
Contig46-orf00925	0.000	0.000	0.000	mycomembrane	QGW23652.1	Putative secreted protein
Contig46-orf03765	0.000	0.000	0.000	Cytoplasmic	QGW25471.1	LSU ribosomal protein L5p (L11e)
Contig46-orf03033	0.000	0.000	0.000	Cytoplasmic	QGW24991.1	Electron transfer flavoprotein, alpha subunit
Contig46-orf05387	0.000	0.002	0.000	Cytoplasmic	QGW26543.1	Leucyl-tRNA synthetase (EC 6.1.1.4)
Contig46-orf02865	0.000	0.000	0.001	Cytoplasmic	QGW24890.1	Malate:quinone oxidoreductase (EC 1.1.5.4)
Contig46-orf04549	0.000	0.002	0.000	mycomembrane	QGW25986.1	hypothetical protein
Contig46-orf03606	0.000	0.001	0.000	Periplasmic	QGW25365.1	Heat shock protein HtrA
Contig46-orf02610	0.000	0.000	0.000	Cytoplasmic	QGW24730.1	Iron-sulfur cluster assembly ATPase protein SufC
Contig46-orf00880	0.000	0.000	0.002	Unknown	QGW23623.1	D-alanyl-D-alanine carboxypeptidase (EC 3.4.16.4)
Contig46-orf00805	0.000	0.000	0.001	Cytoplasmic	QGW23577.1	Acetyl-coenzyme A synthetase (EC 6.2.1.1)
Contig46-orf02634	0.000	0.001	0.000	Cytoplasmic	QGW24748.1	Triosephosphate isomerase (EC 5.3.1.1)
Contig46-orf03006	0.000	0.001	0.001	Cytoplasmic	QGW24975.1	Acetolactate synthase large subunit (EC 2.2.1.6)
Contig46-orf02834	0.000	0.000	0.000	Cytoplasmic	QGW24872.1	Polyribonucleotide nucleotidyltransferase (EC 2.7.7.8)
Contig46-orf03744	0.000	0.001	0.000	Cytoplasmic	QGW25455.1	DNA-directed RNA polymerase alpha subunit (EC 2.7.7.6)
Contig46-orf01451	0.000	0.001	0.000	Cytoplasmic	QGW23967.1	Biotin carboxylase of acetyl-CoA carboxylase (EC 6.3.4.14)
Contig46-orf02978	0.000	0.000	0.001	Cytoplasmic	QGW24957.1	3-isopropylmalate dehydratase large subunit (EC 4.2.1.33)
Contig46-orf02822	0.000	0.000	0.000	Unknown	QGW24865.1	Ribonuclease J2 (endoribonuclease in RNA processing)
Contig46-orf01303	0.000	0.000	0.001	Cytoplasmic	QGW23877.1	Acetyl-coenzyme A synthetase (EC 6.2.1.1)
Contig46-orf04867	0.000	0.000	0.000	Unknown	QGW26201.1	hypothetical protein
Contig46-orf04641	0.000	0.001	0.000	Cytoplasmic	QGW26043.1	3-oxoacyl-[acyl-carrier-protein] synthase, KASIII (EC 2.3.1.180)
Contig46-orf03361	0.000	0.000	0.000	Cytoplasmic	QGW25204.1	3-ketoacyl-CoA thiolase (EC 2.3.1.16) @ Acetyl-CoA acetyltransferase (EC 2.3.1.9)
Contig46-orf00516	0.000	0.000	0.000	Cytoplasmic	QGW23393.1	Putative oxidoreductase YncB
Contig46-orf03189	0.000	0.001	0.000	Extracellular	QGW25092.1	Sporulation protein and related proteins
Contig46-orf01300	0.000	0.001	0.000	Cytoplasmic	QGW23875.1	Citrate synthase (si) (EC 2.3.3.1)
Contig46-orf01341	0.000	0.000	0.000	Cytoplasmic	QGW23898.1	Heat shock protein 60 family chaperone GroEL
Contig46-orf02287	0.000	0.002	0.000	Cytoplasmic	QGW24523.1	SSU ribosomal protein S1p
Contig46-orf03038	0.000	0.000	0.001	Cytoplasmic	QGW24993.1	3-methylmercaptopropionyl-CoA ligase (DmdB)
Contig46-orf01596	0.000	0.001	0.000	Cytoplasmic	QGW24063.1	Ribonucleotide reductase of class Ib (aerobic), alpha subunit (EC 1.17.4.1)
Contig8-orf00025	0.000	0.001	0.000	Unknown	NZ_CP046569.1	putative helicase
Contig46-orf01156	0.000	0.000	0.000	Cytoplasmic	QGW23791.1	Adenylosuccinate lyase (EC 4.3.2.2)
Contig46-orf01480	0.000	0.000	0.000	Cytoplasmic	QGW23989.1	Adenosylhomocysteinase (EC 3.3.1.1)
Contig46-orf01991	0.000	0.000	0.000	Cytoplasmic	QGW24328.1	Aminomethyltransferase (glycine cleavage system T protein) (EC 2.1.2.10)
Contig46-orf02004	0.000	0.001	0.000	Cytoplasmic	QGW24334.1	putative Adenosine kinase (EC 2.7.1.20)
Contig46-orf03097	0.000	0.001	0.000	Cytoplasmic	QGW25031.1	Arginyl-tRNA synthetase (EC 6.1.1.19)
Contig46-orf02563	0.000	0.001	0.001	Cytoplasmic	QGW24700.1	Methylmalonyl-CoA mutase (EC 5.4.99.2)
Contig46-orf05374	0.000	0.000	0.001	mycomembrane	QGW26533.1	hypothetical protein
Contig46-orf02387	0.000	0.001	0.000	Cytoplasmic	QGW24588.1	Glycine dehydrogenase (glycine cleavage system P protein) (EC 1.4.4.2)
Contig46-orf02444	0.000	0.001	0.000	Cytoplasmic	QGW24622.1	Alkylhydroperoxidase protein D
Contig46-orf04929	0.000	0.003	0.000	Cytoplasmic	QGW26248.1	Chaperone protein HtpG
Contig46-orf00012	0.000	0.002	0.000	Cytoplasmic	QGW23080.1	DNA gyrase subunit A (EC 5.99.1.3)
Contig46-orf01916	0.000	0.000	0.001	Cytoplasmic	QGW24281.1	Propionyl-CoA carboxylase beta chain (EC 6.4.1.3) accD6
Contig46-orf05006	0.000	0.001	0.000	Cytoplasmic	QGW26298.1	Medium-chain-fatty-acid--CoA ligase (EC 6.2.1.-)
Contig46-orf04537	0.000	0.000	0.000	Cytoplasmic	QGW25978.1	Fructose-bisphosphate aldolase class II (EC 4.1.2.13)

Contig	H-mMVs-1	H-mMVs-2	H-mMVs-3	Location	Protein ID	Annotation
Contig46-orf00469	0.070	0.081	0.201	mycomembrane	QGW23367.1	putative esterase
Contig46-orf04635	0.105	0.075	0.089	Unknown	QGW26039.1	FIG00995049: hypothetical protein
Contig46-orf04414	0.040	0.069	0.065	mycomembrane	QGW25899.1	putative esterase
Contig46-orf05062	0.037	0.019	0.052	mycomembrane	QGW26334.1	Antigen 85-B precursor (85B)
Contig46-orf05236	0.025	0.044	0.048	mycomembrane	QGW26449.1	hypothetical protein
Contig46-orf03771	0.018	0.020	0.043	Extracellular	QGW25474.1	putative esterase
Contig46-orf00470	0.024	0.019	0.032	mycomembrane	QGW23368.1	putative esterase
Contig46-orf04907	0.010	0.006	0.028	mycomembrane	QGW26230.1	Putative secreted protein
Contig46-orf00456	0.008	0.015	0.026	mycomembrane	QGW23358.1	N-acetylmuramoyl-L-alanine amidase
Contig46-orf04551	0.010	0.032	0.022	mycomembrane	QGW25988.1	No significant database matches
Contig46-orf00641	0.003	0.006	0.021	mycomembrane	QGW23470.1	Putative uncharacterized protein BCG_3873
Contig46-orf04606	0.007	0.005	0.018	mycomembrane	QGW26021.1	Putative serine protease
Contig46-orf04699	0.005	0.007	0.014	mycomembrane	QGW26084.1	possible conserved lipoprotein
Contig46-orf04548	0.004	0.012	0.014	mycomembrane	QGW25985.1	hypothetical protein
Contig46-orf00784	0.002	0.002	0.013	mycomembrane	QGW23561.1	Multimodular transpeptidase-transglycosylase (EC 2.4.1.129) (EC 3.4.-.-)
Contig46-orf02028	0.004	0.001	0.012	mycomembrane	QGW24350.1	putative secreted protein
Contig46-orf01284	0.008	0.004	0.012	Unknown	QGW23866.1	glutamine cyclotransferase
Contig46-orf04415	0.005	0.000	0.011	Unknown	QGW25900.1	hypothetical protein
Contig46-orf04204	0.002	0.006	0.011	Extracellular	QGW25766.1	hypothetical protein
Contig46-orf03658	0.010	0.005	0.010	mycomembrane	QGW25397.1	Phage peptidoglycan binding endopeptidase
Contig46-orf01151	0.023	0.019	0.010	mycomembrane	QGW23786.1	arabinan endo-1,5-alpha-L-arabinosidase A precursor
Contig46-orf03780	0.012	0.002	0.010	Cytoplasmic	QGW25482.1	LSU ribosomal protein L2p (L8e)
Contig46-orf04700	0.010	0.016	0.010	mycomembrane	QGW26085.1	putative lipoprotein
Contig46-orf00647	0.001	0.005	0.009	mycomembrane	QGW23474.1	Trypsin delta/gamma precursor (EC 3.4.21.4)
Contig46-orf00729	0.003	0.004	0.008	mycomembrane	QGW23532.1	hypothetical protein
Contig46-orf04550	0.001	0.003	0.008	mycomembrane	QGW25987.1	hypothetical protein
Contig46-orf04887	0.005	0.005	0.008	mycomembrane	QGW26217.1	hypothetical protein
Contig46-orf04604	0.002	0.014	0.006	mycomembrane	QGW26020.1	putative protease
Contig46-orf02580	0.002	0.002	0.006	Cytoplasmic	QGW24711.1	hypothetical protein
Contig46-orf05343	0.006	0.012	0.006	Periplasmic	QGW26515.1	Maltose/maltodextrin ABC transporter, substrate binding periplasmic protein MalE
Contig46-orf02292	0.006	0.029	0.006	mycomembrane	QGW24525.1	hypothetical protein
Contig46-orf02524	0.005	0.008	0.006	mycomembrane	QGW24679.1	hypothetical protein
Contig46-orf01366	0.004	0.001	0.005	Unknown	QGW23915.1	No significant database matches
Contig46-orf04818	0.004	0.006	0.005	mycomembrane	QGW26166.1	FIG00547498: hypothetical protein
Contig46-orf02020	0.000	0.002	0.005	CytoplasmicMembrane	QGW24345.1	ubiquinol cytochrome C oxidoreductase, cytochrome C1 subunit
Contig46-orf04549	0.001	0.002	0.005	mycomembrane	QGW25986.1	hypothetical protein
Contig46-orf02225	0.015	0.002	0.005	mycomembrane	QGW24478.1	Phage peptidoglycan binding endopeptidase
Contig46-orf03606	0.001	0.000	0.005	Periplasmic	QGW25365.1	Heat shock protein HtrA
Contig46-orf04151	0.008	0.018	0.005	mycomembrane	QGW25732.1	predicted hydrolase or acyltransferase
Contig46-orf02296	0.002	0.015	0.004	mycomembrane	QGW24526.1	hypothetical protein
Contig46-orf00728	0.007	0.009	0.004	mycomembrane	QGW23531.1	Allergen V5/Tpx-1 family protein
Contig46-orf04522	0.001	0.004	0.004	mycomembrane	QGW25968.1	hypothetical protein
Contig46-orf02019	0.002	0.003	0.004	CytoplasmicMembrane	QGW24344.1	Ubiquinol-cytochrome C reductase iron-sulfur subunit (EC 1.10.2.2)
Contig46-orf03752	0.004	0.002	0.003	mycomembrane	QGW25460.1	probable lipase
Contig46-orf00497	0.001	0.001	0.003	mycomembrane	QGW23380.1	probable triacylglycerol lipase(EC:3.1.1.3)
Contig3-orf00065	0.000	0.002	0.003	mycomembrane	NZ_CP046568.1	putative involved in replication/partition
Contig46-orf02583	0.013	0.000	0.003	Cytoplasmic	QGW24713.1	Aconitate hydratase (EC 4.2.1.3) @ 2-methylisocitrate dehydratase (EC 4.2.1.99)
Contig46-orf02266	0.002	0.003	0.003	Extracellular	QGW24503.1	hypothetical protein
Contig46-orf04646	0.002	0.002	0.003	Unknown	QGW26046.1	Copper metallochaperone, bacterial analog of Cox17 protein
Contig46-orf00406	0.000	0.002	0.002	Unknown	QGW23324.1	hypothetical protein

Contig46-orf00095	0.002	0.001	0.002	mycomembrane	QGW23126.1	hypothetical protein
Contig46-orf04582	0.007	0.009	0.002	mycomembrane	QGW26008.1	putative serine/threonine protein kinase (putative secreted protein)
Contig46-orf01560	0.002	0.000	0.002	mycomembrane	QGW24039.1	hypothetical protein
Contig46-orf02030	0.001	0.000	0.002	Periplasmic	QGW24352.1	NLP/P60 family protein
Contig46-orf00361	0.003	0.000	0.002	Cytoplasmic	QGW23292.1	Cysteine synthase (EC 2.5.1.47)
Contig46-orf03789	0.006	0.006	0.002	Cytoplasmic	QGW25488.1	Translation elongation factor Tu
Contig46-orf02610	0.001	0.000	0.002	Cytoplasmic	QGW24730.1	Iron-sulfur cluster assembly ATPase protein SufC
Contig46-orf00794	0.002	0.001	0.002	Cytoplasmic	QGW23569.1	cAMP-binding protein
Contig46-orf04713	0.003	0.003	0.002	mycomembrane	QGW26095.1	Lyzozyme M1 (1,4-beta-N-acetylmuramidase) (EC 3.2.1.17)
Contig46-orf05111	0.028	0.018	0.002	mycomembrane	QGW26366.1	Triacylglycerol lipase precursor (EC 3.1.1.3)
Contig46-orf03776	0.003	0.003	0.002	Cytoplasmic	QGW25479.1	SSU ribosomal protein S3p (S3e)
Contig46-orf04943	0.006	0.003	0.002	Periplasmic	QGW26255.1	glutamate-binding protein of ABC transporter system
Contig46-orf03066	0.003	0.009	0.002	Cytoplasmic	QGW25011.1	ATP synthase alpha chain (EC 3.6.3.14)
Contig46-orf01276	0.001	0.001	0.001	Unknown	QGW23861.1	FIG00996461: hypothetical protein
Contig46-orf03003	0.005	0.001	0.001	Unknown	QGW24973.1	Ketol-acid reductoisomerase (EC 1.1.1.86)
Contig46-orf04644	0.000	0.000	0.001	Periplasmic	QGW26045.1	Putative Dyp-type peroxidase, associated with bacterial analog of Cox17 protein
Contig46-orf03793	0.001	0.000	0.001	Cytoplasmic	QGW25490.1	SSU ribosomal protein S7p (S5e)
Contig46-orf03759	0.001	0.000	0.001	Cytoplasmic	QGW25466.1	SSU ribosomal protein S5p (S2e)
Contig46-orf05129	0.001	0.002	0.001	mycomembrane	QGW26377.1	BNR repeat domain protein
Contig46-orf01647	0.000	0.007	0.001	CytoplasmicMembrane	QGW24096.1	[Acyl-carrier-protein] acetyl transferase of FASI (EC 2.3.1.38)
Contig46-orf05031	0.004	0.006	0.001	Unknown	QGW26312.1	Glucose-methanol-choline (GMC) oxidoreductase:NAD binding site
Contig46-orf03062	0.007	0.014	0.001	CytoplasmicMembrane	QGW25009.1	ATP synthase beta chain (EC 3.6.3.14)
Contig46-orf04839	0.003	0.000	0.001	mycomembrane	QGW26184.1	secreted lipoprotein, ErfK/YbiS/YcfS/YnhG family
Contig46-orf03895	0.000	0.000	0.001	Cytoplasmic	QGW25556.1	LSU ribosomal protein L11p (L12e)
Contig46-orf00538	0.000	0.000	0.001	mycomembrane	QGW23403.1	5'-nucleotidase (EC 3.1.3.5)
Contig3-orf00073	0.002	0.000	0.001	Periplasmic	NZ_CP046568.1	hypothetical protein
Contig46-orf02007	0.001	0.003	0.001	CytoplasmicMembrane	QGW24337.1	Cytochrome c oxidase polypeptide II (EC 1.9.3.1)
Contig46-orf00880	0.000	0.000	0.001	Unknown	QGW23623.1	D-alanyl-D-alanine carboxypeptidase (EC 3.4.16.4)
Contig46-orf02062	0.000	0.000	0.001	CytoplasmicMembrane	QGW24376.1	Cell division protein FtsI [Peptidoglycan synthetase] (EC 2.4.1.129)
Contig46-orf01876	0.001	0.001	0.001	mycomembrane	QGW24250.1	hypothetical protein
Contig46-orf03129	0.002	0.001	0.001	Extracellular	QGW25054.1	hypothetical protein
Contig46-orf02995	0.005	0.001	0.001	Cytoplasmic	QGW24969.1	D-3-phosphoglycerate dehydrogenase (EC 1.1.1.95)
Contig46-orf04534	0.006	0.006	0.001	Extracellular	QGW25976.1	secreted alkaline phosphatase
Contig46-orf01987	0.000	0.012	0.001	mycomembrane	QGW24326.1	L-asparaginase (EC 3.5.1.1)
Contig46-orf01395	0.041	0.001	0.001	Cytoplasmic	QGW23933.1	O-acetylhomoserine sulfhydrylase (EC 2.5.1.49)
Contig46-orf00925	0.000	0.000	0.001	mycomembrane	QGW23652.1	Putative secreted protein
Contig46-orf00805	0.000	0.001	0.001	Cytoplasmic	QGW23577.1	Acetyl-coenzyme A synthetase (EC 6.2.1.1)
Contig3-orf00056	0.000	0.001	0.001	mycomembrane	NZ_CP046568.1	hypothetical protein
Contig46-orf03792	0.012	0.003	0.001	Cytoplasmic	QGW25489.1	Translation elongation factor G
Contig46-orf02612	0.001	0.000	0.001	Unknown	QGW24732.1	Iron-sulfur cluster assembly protein SufB
Contig46-orf03775	0.002	0.002	0.001	Cytoplasmic	QGW25478.1	LSU ribosomal protein L16p (L10e)
Contig46-orf04994	0.002	0.004	0.001	Unknown	QGW26290.1	Triacylglycerol lipase precursor (EC 3.1.1.3)
Contig46-orf03862	0.005	0.004	0.001	Cytoplasmic	QGW25536.1	DNA-directed RNA polymerase beta subunit (EC 2.7.7.6)
Contig46-orf01420	0.001	0.013	0.001	CytoplasmicMembrane	QGW23947.1	Succinate dehydrogenase flavoprotein subunit (EC 1.3.99.1)
Contig46-orf04633	0.012	0.004	0.001	Cytoplasmic	QGW26037.1	3-oxoacyl-[acyl-carrier protein] reductase (EC 1.1.1.100)
Contig46-orf04365	0.000	0.001	0.001	mycomembrane	QGW25868.1	secreted lipase
Contig46-orf02892	0.002	0.000	0.001	Cytoplasmic	QGW24905.1	SSU ribosomal protein S2p (SAe)
Contig46-orf01212	0.002	0.001	0.001	Periplasmic	QGW23825.1	Phosphate ABC transporter, periplasmic phosphate-binding protein PstS (TC 3.A.1.7.1)
Contig46-orf03014	0.002	0.002	0.001	mycomembrane	QGW24980.1	possible glucose dehydrogenase
Contig46-orf03909	0.003	0.003	0.001	Periplasmic	QGW25565.1	Tricarboxylate transport protein TctC
Contig46-orf04812	0.001	0.007	0.001	CytoplasmicMembrane	QGW26163.1	Putative stomatin/prohibitin-family membrane protease subunit YbbK

Contig46-orf04397	0.009	0.000	0.001	Cytoplasmic	QGW25888.1	Isocitrate lyase (EC 4.1.3.1)
Contig46-orf03765	0.001	0.000	0.000	Cytoplasmic	QGW25471.1	LSU ribosomal protein L5p (L11e)
Contig46-orf03794	0.000	0.000	0.000	Cytoplasmic	QGW25491.1	SSU ribosomal protein S12p (S23e)
Contig46-orf02634	0.001	0.000	0.000	Cytoplasmic	QGW24748.1	Triosephosphate isomerase (EC 5.3.1.1)
Contig46-orf01317	0.000	0.001	0.000	Cytoplasmic	QGW23884.1	LSU ribosomal protein L13p (L13Ae)
Contig46-orf03006	0.002	0.000	0.000	Cytoplasmic	QGW24975.1	Acetolactate synthase large subunit (EC 2.2.1.6)
Contig46-orf02834	0.002	0.001	0.000	Cytoplasmic	QGW24872.1	Polyribonucleotide nucleotidyltransferase (EC 2.7.7.8)
Contig46-orf03954	0.002	0.001	0.000	mycomembrane	QGW25595.1	hypothetical protein
Contig46-orf03033	0.002	0.001	0.000	Cytoplasmic	QGW24991.1	Electron transfer flavoprotein, alpha subunit
Contig46-orf04405	0.003	0.001	0.000	CytoplasmicMembrane	QGW25894.1	hypothetical protein
Contig46-orf00554	0.001	0.003	0.000	Unknown	QGW23411.1	hypothetical protein
Contig46-orf03744	0.005	0.000	0.000	Cytoplasmic	QGW25455.1	DNA-directed RNA polymerase alpha subunit (EC 2.7.7.6)
Contig46-orf02149	0.005	0.001	0.000	Periplasmic	QGW24429.1	periplasmic binding protein
Contig46-orf05148	0.006	0.000	0.000	Cytoplasmic	QGW26389.1	Zinc ABC transporter, inner membrane permease protein ZnuB
Contig46-orf03874	0.000	0.000	0.000	Cytoplasmic	QGW25543.1	MCE-family protein Mce1D
Contig46-orf03348	0.000	0.000	0.000	Unknown	QGW25197.1	possible secreted protein
Contig46-orf05152	0.001	0.000	0.000	Unknown	QGW26391.1	Zinc ABC transporter, inner membrane permease protein ZnuB
Contig46-orf00742	0.000	0.001	0.000	Unknown	QGW23537.1	FIG00863414: hypothetical protein
Contig46-orf02977	0.001	0.000	0.000	Cytoplasmic	QGW24956.1	3-isopropylmalate dehydratase small subunit (EC 4.2.1.33)
Contig46-orf01451	0.001	0.000	0.000	Cytoplasmic	QGW23967.1	Biotin carboxylase of acetyl-CoA carboxylase (EC 6.3.4.14)
Contig46-orf00532	0.001	0.000	0.000	mycomembrane	QGW23400.1	Lipase 1 (EC 3.1.1.3)
Contig46-orf00967	0.000	0.001	0.000	Periplasmic	QGW23677.1	Zinc ABC transporter, periplasmic-binding protein ZnuA
Contig46-orf00035	0.001	0.000	0.000	Cytoplasmic	QGW23093.1	Oxidoreductase, short chain dehydrogenase/reductase family
Contig46-orf02889	0.001	0.000	0.000	Cytoplasmic	QGW24904.1	Translation elongation factor Ts
Contig46-orf03157	0.001	0.000	0.000	Cytoplasmic	QGW25072.1	2,3,4,5-tetrahydropyridine-2,6-dicarboxylate N-succinyltransferase (EC 2.3.1.117)
Contig46-orf03071	0.001	0.001	0.000	CytoplasmicMembrane	QGW25013.1	ATP synthase F0 sector subunit b
Contig46-orf03655	0.003	0.000	0.000	Cytoplasmic	QGW25395.1	Succinyl-CoA ligase [ADP-forming] beta chain (EC 6.2.1.5)
Contig46-orf03860	0.009	0.006	0.000	Cytoplasmic	QGW25535.1	DNA-directed RNA polymerase beta' subunit (EC 2.7.7.6)
Contig46-orf04308	0.000	0.000	0.000	mycomembrane	QGW25831.1	putative hydrolase
Contig46-orf03959	0.000	0.000	0.000	Extracellular	QGW25598.1	LIPOPROTEIN VSAC (FRAGMENT)
Contig46-orf02978	0.001	0.000	0.000	Cytoplasmic	QGW24957.1	3-isopropylmalate dehydratase large subunit (EC 4.2.1.33)
Contig46-orf02822	0.001	0.000	0.000	Unknown	QGW24865.1	Ribonuclease J2 (endoribonuclease in RNA processing)
Contig46-orf02312	0.000	0.000	0.000	Cytoplasmic	QGW24537.1	Translation initiation factor 3
Contig46-orf01868	0.000	0.001	0.000	Unknown	QGW24245.1	EpiH/GdmH-related protein
Contig46-orf01303	0.000	0.000	0.000	Cytoplasmic	QGW23877.1	Acetyl-coenzyme A synthetase (EC 6.2.1.1)
Contig46-orf00418	0.001	0.000	0.000	Cytoplasmic	QGW23333.1	3-hydroxyacyl-CoA dehydrogenase (EC 1.1.1.35)
Contig46-orf04867	0.000	0.001	0.000	Unknown	QGW26201.1	hypothetical protein
Contig46-orf02315	0.001	0.000	0.000	Cytoplasmic	QGW24539.1	LSU ribosomal protein L20p
Contig46-orf04641	0.001	0.000	0.000	Cytoplasmic	QGW26043.1	3-oxoacyl-[acyl-carrier-protein] synthase, KASIII (EC 2.3.1.180)
Contig46-orf03361	0.001	0.000	0.000	Cytoplasmic	QGW25204.1	3-ketoacyl-CoA thiolase (EC 2.3.1.16) @ Acetyl-CoA acetyltransferase (EC 2.3.1.9)
Contig46-orf03340	0.001	0.001	0.000	mycomembrane	QGW25192.1	FIG01000940: hypothetical protein
Contig46-orf03220	0.001	0.001	0.000	Cytoplasmic	QGW25114.1	Isocitrate dehydrogenase [NADP] (EC 1.1.1.42)
Contig46-orf03797	0.000	0.002	0.000	Cytoplasmic	QGW25493.1	Alcohol dehydrogenase (EC 1.1.1.1)
Contig46-orf01442	0.002	0.000	0.000	Periplasmic	QGW23962.1	Ferric iron ABC transporter, iron-binding protein
Contig46-orf05270	0.001	0.002	0.000	CytoplasmicMembrane	QGW26474.1	Multimodular transpeptidase-transglycosylase (EC 2.4.1.129) (EC 3.4.-.-)
Contig46-orf02282	0.001	0.002	0.000	Periplasmic	QGW24518.1	ABC-type amino acid transport system, secreted component
Contig46-orf02810	0.000	0.006	0.000	Cytoplasmic	QGW24858.1	Phage shock protein A (IM30) , suppresses sigma54-dependent transcription
Contig46-orf00363	0.001	0.007	0.000	CytoplasmicMembrane	QGW23293.1	Long-chain-fatty-acid--CoA ligase (EC 6.2.1.3)
Contig46-orf00516	0.001	0.000	0.000	Cytoplasmic	QGW23393.1	Putative oxidoreductase YncB
Contig46-orf01665	0.001	0.000	0.000	Periplasmic	QGW24107.1	ABC transporter component, periplasmic oligopeptide binding protein
Contig46-orf03189	0.000	0.001	0.000	Extracellular	QGW25092.1	Sporulation protein and related proteins

Contig46-orf01484	0.001	0.000	0.000	Unknown	QGW23993.1	LpqB
Contig46-orf02663	0.001	0.001	0.000	Cytoplasmic	QGW24765.1	S-adenosylmethionine synthetase (EC 2.5.1.6)
Contig46-orf01300	0.002	0.000	0.000	Cytoplasmic	QGW23875.1	Citrate synthase (si) (EC 2.3.3.1)
Contig46-orf01589	0.002	0.000	0.000	Periplasmic	QGW24057.1	Beta-galactosidase (EC 3.2.1.23)
Contig46-orf01248	0.002	0.000	0.000	mycomembrane	QGW23845.1	HtaA
Contig46-orf01773	0.002	0.000	0.000	Cytoplasmic	QGW24186.1	Valyl-tRNA synthetase (EC 6.1.1.9)
Contig46-orf01341	0.002	0.000	0.000	Cytoplasmic	QGW23898.1	Heat shock protein 60 family chaperone GroEL
Contig46-orf02287	0.003	0.000	0.000	Cytoplasmic	QGW24523.1	SSU ribosomal protein S1p
Contig46-orf02305	0.001	0.002	0.000	Unknown	QGW24531.1	hypothetical protein
Contig46-orf00396	0.002	0.001	0.000	Cytoplasmic	QGW23315.1	Glutamate synthase [NADPH] large chain (EC 1.4.1.13)
Contig46-orf01974	0.000	0.003	0.000	Cytoplasmic	QGW24317.1	Glutamine synthetase type I (EC 6.3.1.2)
Contig46-orf01456	0.001	0.002	0.000	Cytoplasmic	QGW23971.1	Acetyl-coenzyme A carboxyl transferase alpha chain (EC 6.4.1.2)
Contig46-orf03746	0.002	0.003	0.000	Cytoplasmic	QGW25456.1	SSU ribosomal protein S4p (S9e)
Contig46-orf00932	0.001	0.000	0.000	Cytoplasmic	QGW23656.1	ATP-dependent Clp protease, ATP-binding subunit ClpC
Contig46-orf00484	0.000	0.000	0.000	CytoplasmicMembrane	QGW23374.1	Probable arabinosyltransferase A (EC 2.4.2.-)
Contig46-orf01954	0.000	0.000	0.000	Cytoplasmic	QGW24304.1	Glutamine synthetase type I (EC 6.3.1.2)
Contig46-orf02701	0.000	0.001	0.000	mycomembrane	QGW24786.1	FIG004453: protein YceG like
Contig46-orf01596	0.000	0.001	0.000	Cytoplasmic	QGW24063.1	Ribonucleotide reductase of class Ib (aerobic), alpha subunit (EC 1.17.4.1)
Contig8-orf00025	0.000	0.001	0.000	Unknown	NZ_CP046569.1	putative helicase
Contig46-orf04634	0.001	0.000	0.000	Cytoplasmic	QGW26038.1	Acyl dehydratase
Contig46-orf01156	0.001	0.000	0.000	Cytoplasmic	QGW23791.1	Adenylosuccinate lyase (EC 4.3.2.2)
Contig46-orf01480	0.000	0.000	0.000	Cytoplasmic	QGW23989.1	Adenosylhomocysteinase (EC 3.3.1.1)
Contig46-orf00237	0.000	0.001	0.000	CytoplasmicMembrane	QGW23215.1	Uracil permease
Contig46-orf05033	0.000	0.001	0.000	Cytoplasmic	QGW26314.1	ABC transporter substrate-binding protein
Contig46-orf04407	0.000	0.000	0.000	Cytoplasmic	QGW25895.1	Dihydrolipoamide dehydrogenase (EC 1.8.1.4)
Contig46-orf04423	0.001	0.000	0.000	Cytoplasmic	QGW25904.1	Heat shock protein 60 family chaperone GroEL
Contig46-orf01991	0.001	0.000	0.000	Cytoplasmic	QGW24328.1	Aminomethyltransferase (glycine cleavage system T protein) (EC 2.1.2.10)
Contig46-orf02004	0.001	0.000	0.000	Cytoplasmic	QGW24334.1	putative Adenosine kinase (EC 2.7.1.20)
Contig46-orf03097	0.001	0.000	0.000	Cytoplasmic	QGW25031.1	Arginyl-tRNA synthetase (EC 6.1.1.19)
Contig46-orf03783	0.000	0.000	0.000	Cytoplasmic	QGW25485.1	LSU ribosomal protein L3p (L3e)
Contig46-orf00375	0.000	0.001	0.000	Cytoplasmic	QGW23303.1	Aldehyde dehydrogenase (EC 1.2.1.3)
Contig46-orf00032	0.001	0.000	0.000	CytoplasmicMembrane	QGW23091.1	Alkane-1 monooxygenase (EC 1.14.15.3)
Contig46-orf02097	0.001	0.000	0.000	Unknown	QGW24395.1	Putative secreted protein
Contig46-orf02563	0.000	0.001	0.000	Cytoplasmic	QGW24700.1	Methylmalonyl-CoA mutase (EC 5.4.99.2)
Contig46-orf05374	0.000	0.001	0.000	mycomembrane	QGW26533.1	hypothetical protein
Contig46-orf05387	0.001	0.000	0.000	Cytoplasmic	QGW26543.1	Leucyl-tRNA synthetase (EC 6.1.1.4)
Contig46-orf01657	0.001	0.001	0.000	mycomembrane	QGW24101.1	FIG00545237: hypothetical protein
Contig46-orf04945	0.000	0.001	0.000	CytoplasmicMembrane	QGW26256.1	Glutamate transport ATP-binding protein gluA
Contig46-orf05345	0.000	0.001	0.000	CytoplasmicMembrane	QGW26516.1	Glycerol-3-phosphate ABC transporter, ATP-binding protein UgpC (TC 3.A.1.1.3)
Contig46-orf00804	0.000	0.001	0.000	CytoplasmicMembrane	QGW23576.1	FIG00820727: hypothetical protein
Contig46-orf04864	0.000	0.001	0.000	Cytoplasmic	QGW26198.1	Cyclopropane-fatty-acyl-phospholipid synthase (EC 2.1.1.79)
Contig46-orf01641	0.000	0.001	0.000	CytoplasmicMembrane	QGW24094.1	Sodium-dependent transporter
Contig46-orf02387	0.001	0.000	0.000	Cytoplasmic	QGW24588.1	Glycine dehydrogenase [decarboxylating] (glycine cleavage system P protein) (EC 1.4.4.2)
Contig46-orf01247	0.001	0.000	0.000	Periplasmic	QGW23844.1	hypothetical protein
Contig46-orf04600	0.001	0.000	0.000	Cytoplasmic	QGW26017.1	Chaperone protein DnaK
Contig46-orf02444	0.001	0.000	0.000	Cytoplasmic	QGW24622.1	Alkylhydroperoxidase protein D
Contig46-orf01545	0.001	0.000	0.000	Cytoplasmic	QGW24029.1	Collagen alpha 1(I) chain precursor
Contig46-orf03086	0.000	0.001	0.000	Cytoplasmic	QGW25022.1	Transcription termination factor Rho
Contig46-orf01296	0.001	0.000	0.000	Cytoplasmic	QGW23873.1	Phosphoserine aminotransferase (EC 2.6.1.52)
Contig46-orf05450	0.000	0.002	0.000	CytoplasmicMembrane	QGW26583.1	Inner membrane protein translocase component YidC, long form
Contig46-orf02626	0.002	0.000	0.000	Cytoplasmic	QGW24742.1	Transketolase (EC 2.2.1.1)

Contig46-orf04386	0.002	0.000	0.000	Extracellular	QGW25882.1	DNA-binding protein HU / low-complexity, AKP-rich domain
Contig46-orf02032	0.000	0.001	0.000	Cytoplasmic	QGW24354.1	Long-chain fatty-acid-CoA ligase (EC 6.2.1.3), Mycobacterial subgroup FadD15
Contig8-orf00074	0.002	0.000	0.000	Cytoplasmic	AFY63003.1	Ferredoxin reductase
Contig46-orf01873	0.000	0.002	0.000	CytoplasmicMembrane	QGW24248.1	Beta-propeller domains of methanol dehydrogenase type
Contig46-orf04929	0.002	0.000	0.000	Cytoplasmic	QGW26248.1	Chaperone protein HtpG
Contig46-orf02849	0.001	0.000	0.000	Cytoplasmic	QGW24881.1	Translation initiation factor 2
Contig46-orf04653	0.000	0.002	0.000	Unknown	QGW26050.1	hypothetical protein
Contig46-orf04770	0.002	0.000	0.000	Cytoplasmic	QGW26134.1	Diaminobutyrate-pyruvate aminotransferase (EC 2.6.1.46)
Contig46-orf05127	0.000	0.002	0.000	CytoplasmicMembrane	QGW26375.1	Uncharacterized iron-regulated membrane protein; Iron-uptake factor PiuB
Contig46-orf00609	0.000	0.002	0.000	mycomembrane	QGW23447.1	beta-lactamase domain protein
Contig46-orf03911	0.000	0.002	0.000	CytoplasmicMembrane	QGW25567.1	Tricarboxylate transport membrane protein TctA
Contig46-orf00412	0.001	0.001	0.000	Cytoplasmic	QGW23329.1	hypothetical protein
Contig46-orf00365	0.000	0.002	0.000	Cytoplasmic	QGW23295.1	Alcohol dehydrogenase (EC 1.1.1.1)
Contig46-orf00832	0.002	0.000	0.000	Cytoplasmic	QGW23594.1	DNA topoisomerase I (EC 5.99.1.2)
Contig46-orf02865	0.001	0.002	0.000	Cytoplasmic	QGW24890.1	Malate:quinone oxidoreductase (EC 1.1.5.4)
Contig46-orf00012	0.002	0.000	0.000	Cytoplasmic	QGW23080.1	DNA gyrase subunit A (EC 5.99.1.3)
Contig46-orf01916	0.000	0.002	0.000	Cytoplasmic	QGW24281.1	Propionyl-CoA carboxylase beta chain (EC 6.4.1.3) accD6
Contig46-orf05006	0.001	0.002	0.000	Cytoplasmic	QGW26298.1	Medium-chain-fatty-acid--CoA ligase (EC 6.2.1.-)
Contig46-orf04505	0.003	0.000	0.000	Cytoplasmic	QGW25957.1	O-acetylhomoserine sulfhydrylase (EC 2.5.1.49)
Contig46-orf02711	0.000	0.002	0.000	Unknown	QGW24792.1	type I phosphodiesterase/nucleotide pyrophosphatase
Contig46-orf00508	0.003	0.000	0.000	CytoplasmicMembrane	QGW23387.1	Alkane-1 monooxygenase (EC 1.14.15.3)
Contig46-orf00500	0.000	0.003	0.000	Unknown	QGW23382.1	putative glycosyltransferase
Contig46-orf01989	0.002	0.001	0.000	Cytoplasmic	QGW24327.1	Cytosol aminopeptidase PepA (EC 3.4.11.1)
Contig46-orf03107	0.003	0.000	0.000	Cytoplasmic	QGW25038.1	DEAD-box ATP-dependent RNA helicase CshA (EC 3.6.4.13)
Contig46-orf02017	0.001	0.003	0.000	CytoplasmicMembrane	QGW24343.1	Ubiquinol--cytochrome c reductase, cytochrome B subunit (EC 1.10.2.2)
Contig46-orf04726	0.004	0.000	0.000	Cytoplasmic	QGW26105.1	Phosphoenolpyruvate carboxykinase [GTP] (EC 4.1.1.32)
Contig46-orf00423	0.004	0.000	0.000	Periplasmic	QGW23335.1	Superoxide dismutase [Mn] (EC 1.15.1.1)
Contig46-orf02456	0.000	0.003	0.000	CytoplasmicMembrane	QGW24630.1	NADH dehydrogenase (EC 1.6.99.3)
Contig46-orf03540	0.002	0.002	0.000	Cytoplasmic	QGW25326.1	Sphingolipid ceramide N-deacylase
Contig46-orf03068	0.000	0.004	0.000	Unknown	QGW25012.1	ATP synthase delta chain (EC 3.6.3.14)
Contig46-orf03646	0.000	0.004	0.000	Unknown	QGW25389.1	Indolepyruvate ferredoxin oxidoreductase, alpha and beta subunits
Contig46-orf04608	0.000	0.004	0.000	CytoplasmicMembrane	QGW26022.1	Iron-sulphur-binding reductase
Contig46-orf04628	0.004	0.000	0.000	Cytoplasmic	QGW26034.1	3-methylmercaptopropionyl-CoA dehydrogenase (DmdC)
Contig46-orf04537	0.004	0.000	0.000	Cytoplasmic	QGW25978.1	Fructose-bisphosphate aldolase class II (EC 4.1.2.13)
Contig46-orf05100	0.000	0.004	0.000	CytoplasmicMembrane	QGW26360.1	PTS system, fructose-specific IIA component (EC 2.7.1.69)
Contig46-orf02637	0.004	0.001	0.000	Cytoplasmic	QGW24750.1	NAD-dependent glyceraldehyde-3-phosphate dehydrogenase (EC 1.2.1.12)
Contig46-orf01781	0.005	0.000	0.000	Cytoplasmic	QGW24190.1	Ribonuclease E (EC 3.1.26.12)
Contig46-orf03119	0.001	0.004	0.000	Cytoplasmic	QGW25046.1	2-oxoglutarate dehydrogenase E1 component (EC 1.2.4.2)
Contig46-orf03448	0.004	0.001	0.000	Cytoplasmic	QGW25269.1	Enolase (EC 4.2.1.11)
Contig46-orf01419	0.001	0.005	0.000	CytoplasmicMembrane	QGW23946.1	Succinate dehydrogenase iron-sulfur protein (EC 1.3.99.1)
Contig8-orf00071	0.006	0.000	0.000	CytoplasmicMembrane	AFY63000.1	putative cytochrome P450 hydroxylase
Contig46-orf03063	0.001	0.005	0.000	Cytoplasmic	QGW25010.1	ATP synthase gamma chain (EC 3.6.3.14)
Contig46-orf01238	0.008	0.000	0.000	Cytoplasmic	QGW23839.1	Enoyl-CoA hydratase [isoleucine degradation] (EC 4.2.1.17)
Contig46-orf02796	0.009	0.000	0.000	Cytoplasmic	QGW24848.1	Malate synthase G (EC 2.3.3.9)
